# Investigating the Relationship of Genotype and Geographical Location on Volatile Composition and Sensory Profile of Celery (*Apium graveolens*)

**DOI:** 10.3390/ijms222112016

**Published:** 2021-11-06

**Authors:** Lucy Turner, Stella Lignou, Frances Gawthrop, Carol Wagstaff

**Affiliations:** 1 Department of Food and Nutritional Sciences, University of Reading, Harry Nursten Building, Whiteknights, Reading RG6 6DZ, UK; L.Turner@pgr.reading.ac.uk (L.T.); c.wagstaff@reading.ac.uk (C.W.); 2 A.L. Tozer Ltd., Pyports, Downside Bridge Road, Cobham KT11 3EH, UK; frances.gawthrop@tozerseeds.com

**Keywords:** celery, aroma, volatile compounds, SPME GCMS, phthalides, terpenes, harvest

## Abstract

Numerous varieties of celery are grown in multiple countries to maintain supply, demand and availability for all seasons; thus, there is an expectation for a consistent product in terms of taste, flavour, and overall quality. Differences in climate, agronomy and soil composition will all contribute to inconsistencies. This study investigated the volatile and sensory profile of eight celery genotypes grown in the UK (2018) and Spain (2019). Headspace analysis determined the volatile composition of eight genotypes, followed by assessment of the sensory profile using a trained panel. Significant differences in the volatile composition and sensory profile were observed; genotype and geographical location both exerted influences. Two genotypes exhibited similar aroma composition and sensory profile in both locations, making them good candidates to drive breeding programmes aimed at producing varieties that consistently display these distinctive sensory properties. Celery samples harvested in the UK exhibited a higher proportion of sesquiterpenes and phthalides, whereas samples harvested in Spain expressed a higher aldehyde and ketone content. Studying the relationship between growing environment and genotype will provide information to guide growers in how to consistently produce a high-quality crop.

## 1. Introduction

*Apium graveolens*, commonly known as celery, is a vegetable with long fibrous stalks, belonging to the Apiaceae or Umbelliferae family, characterised by its discoid or ‘umbrella’-shaped flowers, known as umbels. Similar to other members of the Apiaceae family, including carrots, coriander and parsley, celery possesses a strong, distinct flavour profile, placing it as a key component in soups, stocks and sauces [1,2]. Compounds that constitute the aroma profile include a range of monoterpenes (myrcene, limonene, β-pinene and γ-terpinene), sesquiterpenes (β-caryophyllene, α-humulene, α- and β- selinene) and phthalides (sedanenolide, neocnidilide and 3-n-butylphthalide) [2,3,4,5,6,7]. The latter compounds have been reported throughout the literature to be the characteristic odour compounds of celery [7], with odour characteristics identified by Turner, Dawda, Gawthrop, Wagstaff and Lignou [8] of ‘celery’, ‘cooked celery’ and ‘herbal’. Celery has long been grown and consumed globally and, for this reason, the aroma profile has been studied using a range of cultivars, grown in a variety of years and geographical locations, and analysed using extraction methods including solvent assisted flavour extraction (SAFE) and solid phase microextraction (SPME) which are, most typically, followed by gas chromatography/mass spectrometry (GCMS) [3,4,5,6,8]. Possibly the earliest investigation, completed by Gold and Wilson [9], determined the volatile composition of celery juice using distillation followed by gas chromatography. This identified a collection of compounds ranging from aldehydes, esters, alcohols and, most importantly, phthalides. More recently completed work not only confirms the compounds identified by Gold and Wilson [9] but displays the complex aroma profile of celery and the variety of compound groups that comprise the aroma profile [7].

As a commonly used vegetable, there is an expectation for celery to be available continuously for consumers; however, in countries such as the United Kingdom, this is not possible due to the unfavourable winter conditions. During the summer months, celery can be grown in the UK as the environment is suitable for growth and, often, celery can continue to be grown on the east coast through autumn. Nevertheless, the annual consumer demand for celery is not met. To combat this issue, celery is grown in warmer locations, such as southern Spain, where they are packaged and processed and then transported to UK retailers. Although offering a solution to meet the demand, utilising seasons in Spain means growing in arid and semi-arid conditions, requiring different agronomy compared to that needed for the UK’s growing environment, and thus creating inconsistencies within the aroma quality of the celery produce available. While not thoroughly understood within celery, the influence of abiotic and biotic factors upon the aroma of crops in general has been investigated by others, and differences have been observed [7,10,11,12,13]. Exposure to different stresses such as temperature, relative humidity, soil and water compositions have been shown to influence the production of primary and secondary metabolites, ultimately leading to variation within the volatile composition [7,10]. Previously, Turner, Lignou, Gawthrop and Wagstaff [10] observed significant differences in the volatile composition and sensory profile of eight celery genotypes grown in the same geographical location in 2018 and 2020. Despite the genotypes displaying significant interactions, it was the differences in environment over the two seasons that had a stronger influence over the volatile composition of celery. The review recently completed by the authors [7] combined data from previously published experiments that investigated the aroma profile of celery, identifying missing data through the exclusion of information, including cultivar name, origin, location of growth, harvest year and conditions of growth. Exposing variation in the presence or absence of compounds and their composition within celery, the authors concluded that without stating all experimental information, the data became unrepeatable. To overcome this, the authors put forward the Minimum Information About a Plant Aroma Experiment (MIAPAE), inviting authors to include parameters used during preharvest, harvest and postharvest as well as extraction and analysis methods, allowing for the building of a repository whereby aroma data for plants can repeated and interpreted correctly [7].

Albeit limited, investigations exploring the impact of geographical locations on celery have been completed; Marongiu et al. [11] compared the volatile composition of wild celery grown and collected in Portugal and Italy as well as using different extraction methods (super critical fluid extraction and hydrodistillation). Differences in the composition caused by both the geographical location and extraction method were observed. Phthalide compounds including sedanenolide and neocnidilide expressed significant differences according to these factors, ultimately concluding that environmental differences between Portugal and Italy were the main cause of observed compositional differences. The cultivar of the wildtype celery used in this study was not included, nor were differences in agricultural techniques and growing environments. However, observed variances in the aroma composition in celery caused by these factors have previously been displayed. Rożek, Nurzyńska-Wierda and Kosior [12] identified that drought stress led to an increase in essential oil due to an increase in the production of secondary metabolites, whereas van Wassenhove, Dirinck, Schamp and Vulsteke [13] observed changes in the phthalide and terpene content when nitrogenous fertiliser (organic and/or inorganic) was applied to celery.

This study aims to investigate the relationship between genotype and geographical location of cultivation upon the volatile composition of eight celery varieties grown in Ely, UK in 2018 and Aguilas, Spain in 2019. Sensory evaluation using a trained panel was completed to understand how chemical and physiological changes lead to differences in the organoleptic perception and to identify interactions between compound groups and geographical location. Ultimately, this information can be used to assist breeders and growers to develop and select cultivars that are optimal for specific growing environments, to produce a consistently flavoured product. Although factors such as temperature and relative humidity are uncontrollable, growers can apply organic/inorganic fertilisers, herbicides/fungicides and supplementary irrigation to aid optimal conditions for celery growth. 

## 2. Materials and Methods

### 2.1. Celery Material and MIAPAE Standard

#### 2.1.1. Sample Information

The eight parental celery genotypes used in these field trials were chosen due to their differences in physical and chemical attributes. Although commercial confidentiality precludes revealing the exact genetic identity of each line used in this paper, the origins of these parental breeding lines and their image postharvest can be found in Supplementary Material (Table S1). Prior to GC/MS analysis, celery material was freeze-dried to ensure consistent aroma quality throughout instrumental analysis. As expected, volatile loss was observed between fresh and freeze-dried samples, however, consistency in relative amount was observed throughout repetitions and the most reported compounds were also identified. Freeze-drying is a method that has been used previously to preserve the volatile content of herbs [14,15,16], and, furthermore, Hoffman [17] identified freeze-drying as a preservation method that best retains a typical aroma at a strong intensity.

#### 2.1.2. Timing, Location and Environment

Celery seed (*Apium graveolens*) of eight parental genotypes supplied by Tozer Seeds Ltd. (Cobham, United Kingdom) were grown in commercial conditions and harvested in Cambridgeshire (United Kingdom) by G’s Fresh Ltd. (Ely, United Kingdom (52°21′12.9″ N 0°17′15.6″ E)) during spring/summer 2018. In 2019, the same eight parental varieties of celery were grown and harvested in Aguilas, Spain by G’s España Ltd. (37°25′43.2″ N 1°39′56.2″ W).

Celery grown in the UK was grown on sandy loam soils with naturally high groundwater and a peaty surface, whereas celery grown in Spain was grown on Calcisol soils. Both harvests were grown in a randomised block design, using commercial celery products as border plants to remove edge effects and subjected to the same commercial conditions including application of agronomic techniques, fertilizer and irrigation as commercial celery. For both years, 20–25 mm of overhead irrigation was used every four days, and standard commercial fertiliser, pest and disease control regimes were applied. In 2018, plugs were transplanted mid-June after 22 days’ growth in the nursery, then harvested 91 days later. The average daily air temperature was 18.2 °C, with 0.2 mm of rainfall daily and an average relative humidity of 88.1%. Average wind speed was 1.9 ms and the dew point was 15.5 °C. In 2019, plugs were transplanted in early January after growing for 20 days in the nursery, then harvested in late March, 87 days later. The average daily air temperature was 17.6 °C, with 0.4 mm of average rainfall and an average relative humidity of 77.3%. Average wind speed was 1.7 ms and dew point was 6.0 °C. Prior to harvest, the celery was subject to regular in-field assessment to ensure standards for commercial quality were met, including visual and taste tests. These celeries were harvested within a close timeframe of the commercial produce also being grown in the field, which acted as an indicator for the appropriate commercial harvest maturity.

#### 2.1.3. Raw Material Collection, Processing Storage

The celery was grown at a density of 10 plants m^−2^, and three replicates were harvested from each block using a celery knife. Celery petioles were cut to 20 cm, discarding outer petioles, the base, leaves and any knuckles, and sealed in labelled bags for transportation to the University of Reading (United Kingdom). Harvesting in Spain followed the same procedure; however, celery was packed into cool boxes and transported to the UK in refrigerated conditions using G’s Fresh Ltd. courier. Transportation took two days and samples were collected from G’s Fresh (Ely, Cambridgeshire, UK) before transportation back to the University of Reading. 

Celery samples used for sensory evaluation were refrigerated for one day before presenting to the trained panel, whereas samples for aroma analysis were immediately frozen at −80 °C for one week and subsequently freeze-dried for five days. Samples were then milled to a fine powder using a milling machine (Thomas Scientific, Swedesboro, NJ, USA) and stored in an airtight container for a maximum of two weeks before analysis with gas chromatography/mass spectrometry (GC/MS).

### 2.2. Chemicals Reagents

For GC/MS analysis, calcium chloride and the alkane standard C_6_–C_25_ (100 μg mL^−1^) in diethyl ether were obtained from Merck (Poole, UK).

### 2.3. Volatile Analysis Using SPME GCMS

For headspace sampling, the celery sample (0.5 g) was combined with 0.5 mL of saturated calcium chloride solution and filled to 5 mL using HPLC-grade water in a 15 mL SPME vial fitted with a screw cap. Samples were analysed by automated headspace SPME using an Agilent 110 PAL injection system and Agilent 7890 gas chromatograph with 5975C mass spectrometer (Agilent, Santa Clara, CA, USA) according to Turner et al. [8,10].

### 2.4. Sensory Evaluation of Fresh Celery Samples

Sensory evaluation was carried out using quantitative descriptive analysis (QDA^TM^) to determine the sensory characteristics of the eight celery samples, and the characteristics were estimated quantitatively. The trained sensory panel at the Sensory Science Centre (University of Reading, n = 12; 11 female and 1 male) was used to develop a consensus vocabulary to describe the sensory characteristics of the eight celery genotypes. The terms were discussed by the panellists as a group, facilitated by a panel leader, and this led to a consensus of 22 and 23 attributes for the UK and Spanish harvest, respectively. The sensory assessment of the samples was carried out according to Turner et al. [8] at the Sensory Science Centre (University of Reading) using Compusense Cloud Software (Version 21.0.7713.26683, Compusense, Guelph, ON, Canada) to acquire the data.

### 2.5. Statistical Analysis

The percentage composition was calculated from the peak area data collected by SPME GC/MS analysis, and quantitative data for each compound identified in the SPME GC/MS analysis were analysed by both one- and two-way analysis of variance (ANOVA) and principal component analysis (PCA) using XLSTAT Version 2020.1.3 (Addinsoft, Paris, France). For those compounds exhibiting significant difference in the one-way ANOVA, Tukey’s honest significant difference post hoc test was applied to determine which sample means differed significantly (*p* < 0.05) between geographical location and the celery genotypes. Only those compounds exhibiting significant differences between geographical location (G), genotype (*E*) and their interaction (GxE) were included in the PCA. 

SENPAQ version 6.3 (Qi Statistics, Kent, UK) was used to carry out the ANOVA of sensory panel data. The means from sensory data were taken over two sessions for all assessors and correlated with the percentage composition means from the instrumental data via PCA using XLSTAT. 

## 3. Results and Discussion

### 3.1. Volatile Composition

In total, 118 compounds were detected in the headspace of the eight celery genotypes in both geographical locations (UK and Spain) (Table 1). Sixty-five compounds were identified in 2018 across eight genotypes, including: 22 monoterpenes, ten sesquiterpenes, eight aldehydes, five alcohols (three of which are classified as monoterpenoid alcohols) and five phthalides. Additional compounds were identified in the headspace of the same genotypes from the Spanish harvest including: 27 monoterpenes, 17 aldehydes, 11 sesquiterpenes and alcohols (six of which are classified as monoterpenoid alcohols), nine ketones and six phthalides. Quantitative differences were observed between the two geographical locations as well as the eight genotypes in this study, and two-way ANOVA revealed significant differences in aroma difference caused by both factors. Where Spanish grown celery displayed higher alcohol, aldehyde and ketone content, UK grown celery expressed a much higher monoterpene, sesquiterpene and phthalide content. Seventeen compounds expressed no significant difference in relative amount by these factors and seven of these came from lower boiling compounds, including camphene, sabinene and β-pinene, along with D-carvone and carvacrol. These low boiling monoterpenes were not observed to differ significantly when harvested in 2018 and 2020 in the UK [10], suggesting that monoterpenes are fundamental to the crop and factors including genotype and climate hold limited influence over the abundance of these compounds.

As observed in various studies, monoterpenes, sesquiterpenes and phthalides are the most reported compound groups to contribute to celery’s aroma profile [4,5,6,8,36,37]. The composition of celery grown in UK expressed an average of 55% monoterpenes, 20% phthalides and 9.2% sesquiterpenes, whereas genotypes grown in Spain had an average of 32%, 2.2% and 9%, respectively. Monoterpenes comprised most of the composition of the aroma profile of all celery genotypes grown in the UK, with limonene, γ-terpinene, β-pinene and *m*-cymene exhibiting the highest proportion of monoterpenes [4,7]. A lower proportion of monoterpenes comprised Spanish-grown celery, however, genotypes 10 and 12 displayed over 10% more than the other genotypes (Table 1). The authors previously carried out gas chromatography–olfactometry (GC/O) on two celery genotypes (12 and 25) and reported that these compounds contribute citrus, fresh, pine, and mint odours to celery [8]. Although these compounds comprised much of the aroma profile, their odour activity remains low and, therefore, they would not be considered characteristic compounds to celery. By completing aroma extraction dilution analysis (AEDA), Kurobayashi, Kouno, Fujita, Morimitsu and Kubota [38] identified the flavour dilution (FD) factor of volatile compounds of raw and boiled celery. Phthalides including 3-n-butylphthalide and ligustilide were found to have the highest FD factor of 3125, whereas myrcene, a monoterpene also identified within the current study, had a FD value of 625. Uhlig, Chang and Jen [3] investigated the effect of phthalides on celery flavour using eight celery cultivars of varying origins, observing a positive correlation with total phthalide content and the intensity of the ‘celery flavour’ attribute. Significant variation between celery cultivars and phthalide content was also observed, most obviously in the concentration of sedanenolide. This is reflected in the current study.

The prominence of phthalides and their contribution to celery aroma is apparent throughout literature. A review completed by the authors [7] identified 3-n-butylphthalide and sedanenolide to be the most reported phthalides in celery, with odour descriptors such as celery, herbal and cooked celery. These compounds have been identified as characteristic compounds to celery aroma, and when authors [8] completed GC/O upon two celery genotypes also used in this study (12, 22), the average odour intensity of these compounds was high throughout maturity. Growing celery in the UK in 2018 produced genotypes with a higher phthalide composition, particularly high in 3-n-butylphthalide and sedanenolide, comprising an average percentage of 7.1% and 11.6%, respectively. The average percentage of these compounds was lower in celery growing in Spain in 2019, with 3-n-butylphthalide and sedanenolide contributing an average of 5.3% and 2.6%, respectively. However, (*Z*)-neocnidilide was expressed at a higher composition in Spanish celery, comprising an average of 0.92% of the aroma profile. Pino, Rosado and Fuentes [39] identified sedanenolide to comprise much of the volatile profile of celery leaf oil, comprising 32.1% of the composition. The significantly higher abundance of these phthalide compounds, reflected in Table 1, will allow assumptions to be drawn that these genotypes have a stronger typical celery aroma [3].

A similar pattern was observed within sesquiterpenes, whereby celery grown in the UK exhibited a significantly higher proportion of sesquiterpenes compared to Spanish grown celery. β-Caryophyllene and β-selinene comprised the highest proportion of the sesquiterpene profile for both geographical locations, and these two are the most reported sesquiterpenes in celery [7,36,37,40]. A similar sesquiterpene trend was observed in another study [10] between two harvest years (2018 and 2020) for the same eight genotypes, whereby the sesquiterpene content comprised a higher proportion of the volatile profile of celery grown in 2018, a significantly warmer season than 2020 [10]. Pino, Rosado and Fuentes [39] identified β-caryophyllene to comprise 13.5% of the volatile profile of Cuban celery leaf oil, whereas Lund, Wagner and Bryan [41] identified β-caryophyllene and β-selinene to comprise an average of 1.5% and 3.4%, respectively. Lund, Wagner and Bryan [41] also identified β-selinene to have a celery-like odour.

Whilst monoterpenes formed much of the composition of UK grown celery, aldehydes were observed to contribute a high proportion in Spanish-grown celery for all genotypes, except genotypes 10 and 12, comprising an average of 38.5% of the aroma composition. Hexanal and (*E*)-2-heptenal were the most abundant compounds in this group for both geographical locations and genotypes, with odour characteristics of fresh, green and fatty. Although not identified in UK grown celery, benzaldehyde and (*E*)-2-octenal composed a high proportion of the volatile composition, with odour characteristics of almond, cherry, and cucumber, averaging to comprise 2.0% and 2.7%, respectively. Aldehyde content within celery has not been discussed thoroughly, with only a few studies detecting the compound group. Gold and Wilson [9] identified a range of aldehydes including hexanal, octanal and heptanal, yet Shojaei, Ebrahimi and Salimi [40] only identified benzeneacetaldehyde and nonanal within three ecotypes of wild celery. A large proportion of aldehydes that were identified in the current study were detected, using GC/O, to be prominent throughout celery maturity [8]. Hexanal was one of the compounds contributing the most to the aldehyde content in celery for all genotypes across both locations, with odour characteristics including fresh, green and apple, as well as being identified throughout celery maturity [8].

Similarly, the ketone content of celery has rarely been discussed and only few studies have reported these compounds [8,9,40]. Accompanying the identification of aldehydes, Shojaei, Ebrahimi and Salimi [40] further detected *p*-methyl acetophenone and 2-undecanone within the three wild celery ecotypes. An explanation for the variation in ketone content between geographical location could involve investigating the formation of phthalides. The metabolic pathway involved in the synthesis of phthalides has yet to be confirmed and, currently, there are multiple suggestions looking into how phthalides are synthesised [7]. Phan, Kim, and Dong [42] identified a method of synthesising phthalides through ketone hydroacylation. Here, the hydroacylation of ketones led to the formation of five-membered lactones, inducing the synthesis of 1(3H)-isobenzofuranone, the simplest phthalide structure. From here, various phthalides can be formed according to the substitution at C3 [7,42]. The large variety of ketones identified (Table 1) may be an indication of the potential for the Spanish crop to synthesise phthalides. Many ketones were identified by the authors [8] to be important to celery aroma when using GC/O to measure the change in aroma during celery maturity. The compounds 3-Pentanone, 2-hexanone and 3-octen-2-one were detected at higher intensities in immature celery, displaying the crop’s potential to synthesis phthalide compounds, whereas 1-octen-3-one was identified by GC/MS with a relative abundance of 6.7 and 4.7 AU, respectively, in post-mature celery.

#### Principal Component Analysis of Volatile Compounds in UK and Spanish Celery Samples

**Figure 1:**
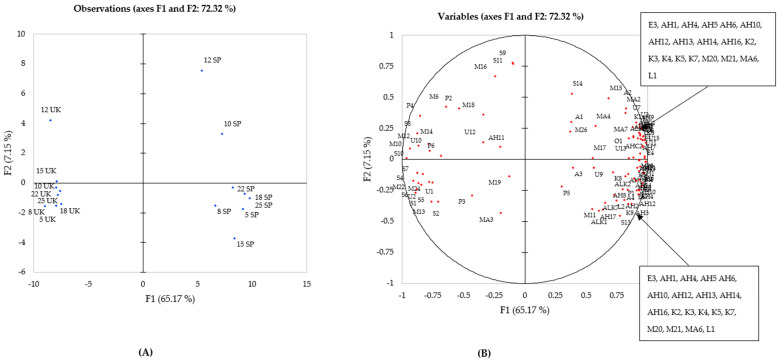
Principal component analysis of eight celery samples harvested in the UK in 2018 and Spain in 2019 showing correlations with volatile compounds. (**A**) Projection of the samples; (**B**) Distribution of variables.

Principal component analysis allowed for the visual comparison of the volatile composition of the eight celery genotypes grown in UK and Spain (Figure 1) and to examine any correlations occurring between genotype, geographical location and chemical compounds. Using only the significant compounds for geographical location (G), genotype (*E*) and their interaction (GxE), a clear divide between the compounds associated with each year was observed. Principal component one (F1) and two (F2) explained 72.32% of the total variation present in the data, and it can be observed that the first axis separated samples from the geographical location (UK and Spain), whereas the second axis separated the various genotypes within a location. Differences between geographical location were apparent, as they separated along the F2 component.

Genotype expressed a significant influence over both the UK- and Spanish-grown celery (Table 1), yet a more noticeable separation was observed in the Spanish-grown celery between genotypes, in addition to a strong association with more aroma compounds than UK celery (Figure 1). Genotype expressed significant differences (Table 1), but genotypes 12, 22 and 25 for Spain were positioned in a similar place on the opposite quadrant in the observation plot. Genotype 12 in both locations took the appearance of an outlier, displayed as the most significantly different from other genotypes used within this experiment. This was caused by the high abundance of sesquiterpene compounds present in the UK harvest, especially from β-selinene, and the high phthalide content within the Spanish harvest, with 3-n-butylphthalide and sedanenolide comprising 8.5% and 9.2%, respectively, of the total volatile content. Significant compound associations with Spanish grown celery were expressed within Figure 1 including all aldehydes (except AH11) and ketones, accompanied by monoterpenes (M11, 15, 17, 20, 26), sesquiterpenes (S13, 14), phthalides (P1, 5) and alcohols (A1, 2, 3). This was further reflected in Table 1. Conversely, less noticeable separation between the eight celery genotypes was observed by celery grown in the UK, in addition to fewer compound associations. Monoterpenes (M6, 10, 12, 13, 14, 16, 18, 22, 24), sesquiterpenes (S1, 2, 4, 5, 6, 7, 8, 10, 12) and phthalides (P2, 3, 4, 6) were positively correlated with samples grown in the UK. The spread of monoterpenes, sesquiterpenes and phthalides across the plot, together with ubiquity within all celery genotypes regardless of location of growth, harvest year [10] and maturity [8], confirmed the importance of these compound groups to celery and celery aroma. This was originally concluded by the authors [10], where eight genotypes of celery grown in the UK in 2018 and 2020 both exhibited these compounds, and in a similar pattern. Aldehydes and ketones appeared to be more strongly influenced by geographical location rather than genotype, explaining why these compounds are not commonly reported within the celery volatile composition.

Genotype and geographical location both expressed a significant influence over the volatile content of celery (Table 1), however, geographical location expressed a stronger influence upon the composition (Figure 1). Differences within the growing climate and agronomy applied to the celery increased the risk of variation, as similarly expressed between harvest years [10], whereby differences in air temperatures were likely the cause for the large variation expressed between years 2018 and 2020, altering the sensory profile of the crop. The differences in composition observed between the eight celery genotypes grown in the UK and Spain (Figure 1) and the impact that these have upon the sensory characteristics were investigated through sensory profiling.

### 3.2. Sensory Evaluation of Fresh Celery Samples

The sensory profile of the eight celery samples was generated by a trained panel who came to the consensus of 22 and 23 terms for the quantitative assessment of samples grown in the UK in 2018 and samples grown in Spain in 2019, respectively. The additional attribute for the samples grown in Spain in 2019 was salty taste, and we hypothesised that this was because of the saline soils present in this part of the country, as observed in other studies such as tomato [43], pepper [44] and cauliflower [45]. Mean panel scores for these attributes are presented in Table 2. Out of the 22 attributes that were profiled from the UK harvest, 14 of these were found to be significantly different between the genotypes, and seven out of 23 attributes were significantly different for the Spanish trial in 2019. Few significant assessor x sample interactions were identified for both UK and Spanish harvests, suggesting that the panellists scored samples in a consistent manner [46]. Statistical comparison of sensory differences between locations could not be completed due to the one-year difference between harvests, however, general trends will be discussed.

Appearance attributes for both locations displayed significant differences caused by genotype, and similarities were observed between scoring for stalk thickness and colour attributes. A significant difference (*p* < 0.001) for ribbed appearance was apparent between locations for all genotypes. The genotype variation between ribbed appearance was more apparent for those harvested in the UK than those harvested in Spain, with scores ranging from 25.4 to 65.9. Mouthfeel attributes displayed a positive correlation with appearance attributes, and these attributes were the highest scoring attributes in all genotypes across both locations, apart from stringiness. Stringiness was scored higher in Spanish celery, with all genotypes of the Spanish celery recording an increase of at least 10, apart from genotype 22. Genotype 22 was scored significantly lower for stringiness when comparing other genotypes in both locations. Although not significantly different, grassy after-effect was scored higher within UK celery and exhibited a positive correlation with grassy odour, an attribute that was significantly different in both locations.Significant differences in the odour and flavour attributes evaluated in both genotypes and geographical location were observed but, more significantly, different attributes were identified in UK celery. The cucumber and rocket flavour with grass odour attributes were scored higher in the UK harvest, whereas Spanish-grown celery scored higher for fresh coriander odour, fennel and soapy flavour. The fresh coriander flavour attribute was scored alike for both locations, however genotype 12 displayed a higher score in coriander flavour when grown in Spain, going from a score of 9.6 to 17.4. Furthermore, genotype 12 was scored as most bitter with genotype 8 and 18 for both locations, but scored sweeter when grown in Spain. Genotype 18 was scored with the strongest soapy flavour, which expressed a positive correlation with fresh fennel. Where genotype 12 scored high for flavour/odour attributes (apart from cucumber), genotype 25 scored low for flavour/odour attributes, only scoring high in the cucumber flavour attribute in both locations.

#### Principal Component Analysis of Flavour Attributes and Volatile Compounds

**Figure 2:**
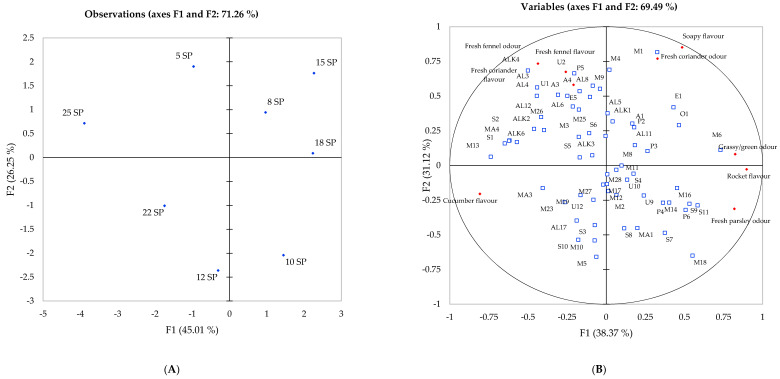
Principal component analysis of eight celery samples harvested in UK 2018 showing correlations with volatile compounds and sensory attributes. (**A**) Projection of the samples; (**B**) Distribution of variables.

**Figure 3:**
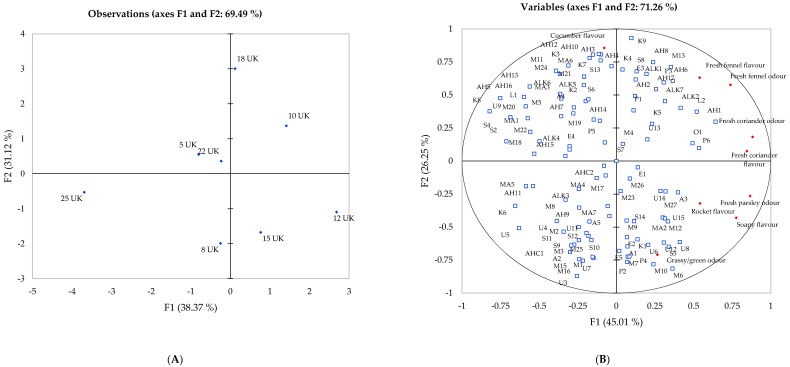
Principal component analysis of eight celery samples harvested in Spain 2019 showing correlations with volatile compounds and sensory attributes. (**A**) Projection of the samples; (**B**) Distribution of variables.

PCA was used to visualise the sensory and chemical differences observed across the eight genotypes, with the volatile compounds identified (Table 1) and the sensory attributes related to odour and flavour used as variables (Figure 2 and Figure 3). Celery grown in the UK expressed a large variation between the eight genotypes (Figure 2), whereby principal component one (F1) and two (F2) explained 69.49% of the total variation within the data. The first axis separated genotypes 5, 10, 18 and 22 from other genotypes, whereas the second axis separated genotypes 10, 12, 15 and 18. Genotype 25 was scored the lowest for all flavour attributes, only scoring high in cucumber flavour (Table 2), whereas genotype 12 opposed genotype 25 (Figure 2) and displayed strong association with a fresh parsley and grass odour along with a rocket flavour. Genotype 18 was positively correlated with fresh fennel and coriander flavour, with the soapy characteristics that accompany many members of the Apiaceae family [47]. A grouping of aroma compounds in the centre of the PCA was observed, whereas the sensory characteristics remained positioned on the outer rim of the biplot, with genotypes 5 and 22 grouped in the middle of the observation plot accompanied with no strong associations with any flavour/odour attribute (Figure 2). These genotypes exhibited a lower volatile content to genotype 12 (Table 1). Predominantly, monoterpenes and sesquiterpenes were negatively correlated with the first principal component (F1), and compounds belonging to compound classes such as alcohols and aldehydes were positively associated with F1. Phthalides were distributed around the plot, with (*Z*)-neocnidilide (P5) displaying positive association to fresh fennel, whereas sedanenolide and (*E*)-ligustilide (P4 and P6) express a positive correlation with fresh parsley.

Principal component one (F1) and two (F2) explained 71.26% of total variation observed within the dataset for the samples grown in Spain, and the first axis separated genotypes 10, 12 and 22, whereas genotypes 5, 12, 22 and 25 are separated along the second axis. Genotype 25 in Spain exhibited a low association to all attributes apart from cucumber flavour, observed in UK 25, and genotype 12 in Spain expressed a significant association to grass odour, as observed in the UK. Furthermore, genotype 18 displayed a positive association with fresh coriander and fennel odour and flavour attributes when grown in Spain and the UK. The perception of genotypes 5, 8, 10, 15 and 22 was observed to change significantly between locations, caused by the chemical compositional changes.

The flavour attribute of cucumber displayed no significant correlations in UK compounds (Figure 2), yet significant correlations between compounds and this attribute were observed with multiple aldehydes (AH3, AH5, AH10, AH12 and AH13) that express odour characteristics such as fatty, cucumber and green (Figure 3). These compounds were not identified in the UK harvest. Compounds identified in UK celery (Figure 2) all displayed association with a flavour/odour attribute of sorts; however, this was not reflected within Spanish-grown celery. Plotto et al. [48] calculated the retronasal and orthonasal activity values for selected terpenes and aldehydes in an orange juice matrix, identifying limonene, β-pinene and γ-terpinene to have the highest thresholds in water and orange juice, whereas hexanal, octanal and nonanal, all aldehydes identified in celery (Table 1), expressed a much lower threshold. Due to the lower proportions of monoterpenes identified in Spanish-grown celery, the flavour characteristics contributed by these aldehydes (green, waxy, cucumber, honey [8]), allowed the panel to detect these more easily. This explains the differences observed in the sensory panel between the celery grown in the UK and in Spain. Furthermore, observed on the factor plot in the bottom left quadrant (Figure 3), a large group of compounds displayed no significant associations with any sensory attribute.

Celery harvested in Spain expressed a different aroma profile when compared to samples harvested in the UK, as observed in the significant difference of the aroma composition (Table 1), and although we cannot compare statistically UK and Spanish genotypes, differences in the scoring of attributes were observed. Genotypes 5, 8 and 15 displayed no association with herbal odour and flavour attributes in the UK (Figure 2) but were scored higher after growing in Spain, where strong associations to fresh fennel, coriander and parsley were displayed (Figure 3). Genotype 12 expressed close association with grass and fresh parsley odours, in addition to sedanenolide and 3-n-butylphthalide, compounds known for their celery odours, and displayed significant positive correlations with grass and parsley odour. On the other hand, genotype 25 expressed the lowest relative content of volatile compounds identified, apart from aldehyde compounds, and was scored with a significantly higher cucumber flavour than any other genotype in both locations. Here, we can assume this genotype does not exhibit a strong characteristic odour in comparison to genotype 12. As both these genotypes performed in a similar manner across locations, we would recommend these genotypes to breeders and fresh produce growers who plan to use the same cultivar across different locations, as they have expressed stability in volatile composition.

### 3.3. Environmental Differences between Geographical Location and Influence on the Aroma Profile

In this study, differences in the volatile composition and sensory profile were observed between eight genotypes and two geographical locations. Previously, Turner et al. [10] used the same genotypes grown in different years in the UK and identified that differences in temperatures (air and soil) played an important role in determining the overall flavour of celery. Environmental data including temperature, rainfall and relative humidity were collected at the nearest weather station to the farm of growth and provided by G’s Fresh UK and Grupo G’s España (Table 3) to compare the differences in the climate of geographical location. These environmental and geographical differences and how they influence the chemical composition of celery are only hypothesized due to the inadequate study of different growing conditions on celery. However, abiotic stresses from factors including temperature, humidity, water and mineral availability have been commonly observed in literature to influence secondary metabolic profiles in plants [49,50,51].

Utilising two seasons for growing and using the same eight genotypes, Turner et al. [10] identified that warmer temperatures had a positive correlation with sesquiterpene and phthalide generation, whereas growing in lower temperatures led to celery with a higher monoterpene content. As similarly discussed by the authors [10], data from two harvests are insufficient when stating any relationships between environment and volatile composition, however, collating the data collected in this investigation, the dataset is completed with eight genotypes in a multi-site and multi-year experiment. Similarities in the chemical profile were observed in genotypes 12, 18, 22 and 25 in how they reacted to being grown in an alternative environment, suggesting that genotype predetermines the protective or coping mechanisms for the crop when exposed to abiotic and biotic stresses.

Celery grown in 2018 in the UK was subjected to temperatures much warmer than considered normal for the UK, and the environmental values do not express any significant differences between geographical location (Table 3) apart from the dew point; UK grown celery was grown in an environment where the average dew point value was 15.5 °C, substantially higher when compared to the 5.7 °C experienced by Spanish-grown celery. The observed dew point temperature indicates the temperature required for the air to cool to reach a relative humidity of 100%. The average daily temperature of UK grown celery is 18.2 °C and much closer to the dew point value, confirming the increased humidity experienced by UK grown celery. Exposure to high dew points promotes the growth of pathogens, inhibiting crop growth and, subsequently, compromising the crop to biotic stresses [52]. Specific stresses such as those caused by a pathogen will cause the crop to prepare a stress response and, additionally, increase the rate of plant-to-plant signalling as a form of communication, perhaps explaining the increased content of monoterpene compounds observed by the UK grown crop (Table 1). Sampaio, Edrada-Ebel and Da Costa [53] studied the influence of environmental factors on the secondary metabolic profile of *Tithonia diversifolia*, observing a variation within the metabolic profile in the leaves and stems, expressing a stronger association with rainfall and humidity levels than with temperature and solar radiation. The primary metabolite content of *Tithonia diversifolia* expressed a strong positive correlation with relative humidity, whereas secondary metabolite content expressed a strong negative correlation with humidity. A similar reaction was observed in the present study, whereby more secondary metabolites in the form of volatile compounds were identified in Spanish grown celery, where relative humidity was lower (Table 3).

Due to minimal differences in the climate data, investigating differences in agriculture, including water and soil composition, must be included in the discussion, as these factors will also influence the flavour outcome. As a consequence of the arid and semi-arid conditions of Aguilas, Spain and the increasing shortage of water for crop irrigation, desalinated seawater is often used in southern regions of Spain [54]. Conversely, the crop irrigation system in place within the UK is by fresh water from a nearby reservoir, supplied by the river Little Ouse, in this instance. Although rigorous pre-treatment processing and filtration steps would have been completed upon both water supplies, the mineral composition of water will be vastly diverse due to differences in the original source. This will lead to variances in the soil for uptake in minerals such as calcium, sodium, magnesium, zinc and iron.

Growing in different geographical locations involves growing on different soil types. This will lead to differences in the soil properties including water holding capacity and mineral composition. UK celery was grown on loamy and sandy soils with naturally high groundwater, allowing for high water availability and nutrient uptake, whereas the Calcisol soils of Spain are known for their accumulation of calcium carbonate from precipitation brought about by evaporation under arid and semi-arid conditions [55]. The presence of surplus calcium carbonate in the soil could ultimately cause a stress response by the crop. To promote healthy growth, the crop must uptake soil, waterborne micronutrients and inorganic elements which are necessary for functional growth and involved in an array of essential pathways, including the synthesis of secondary metabolites such as isoprenoid through the non-mevalonate pathway, i.e., the building block for monoterpenes and sesquiterpenes. Primarily, carbon-, nitrogen-, sulphur- and phosphorous-fixation is involved in the synthesis of substrates and precursors involved in primary and secondary metabolism [56]. The micronutrient and element content of the soil and its permeability will influence the uptake of water and minerals from the soil to be utilised within the crop. These micronutrients can be applied by the plant for a range of uses; for example, copper has been identified to improve the flavour of fruits and vegetables along with increasing sugar and lignin content, zinc promotes the transformation and consumption of carbohydrates in plants and iron is a prominent micronutrient involved in the synthesis of organic acids [57,58]. Applying fertilisers (organic or inorganic) will increase the soil micronutrient content leading to the desired elements being available for crop uptake. Calcium and boron deficiencies, known causes of black heart and hollow stem in celery, are both nutrient-deficient illnesses that can be avoided through the application of appropriate sprays and fertiliser [59]. However, van Wassenhove, Dirinck, Schamp and Vulsteke [12] identified the negative impact of using nitrogen-based fertilizer on celery and its volatile composition. Contrary to what has been discussed above, an increased application of a nitrogen fertilizer (organic and/or mineral nitrogen) led to a reduction in the aroma-determining compounds in two celery cultivars. In fact, applying no fertilizer resulted in a higher content of volatile compounds including phthalides, whereas an overall decrease was observed between 1000 and 2000 μg kg^−1^ of fresh material when a nitrogen fertilizer was applied. D’Antuono, Neri and Moretti [60], similarly, observed a decrease in volatile content as nitrogen fertilizer volume was increased, especially in compounds such as limonene, myrcene and β-selinene. However, total phthalide content along with β-caryophyllene and α-selinene were identified in high proportions when 300 kg ha^−1^ of nitrogen was used on celery. It is possible that Spanish grown celery was exposed to higher levels of nitrogen, thus leading to a lower proportion of monoterpenes, sesquiterpenes and phthalides within the aroma composition.

Factors that accompany field placement will be a less significant cause of variation, but when these factors are combined, they will play a more significant role in determining the secondary metabolite content in celery. Possibly the most obvious difference between geographical location would be the altitude of each field: UK celery was grown on an east-facing field that was −1 to 1 m above sea level, whereas the field in Aguilas was south-facing at 390 m above sea level. Higher altitudes will result in lower temperatures and limitations on light exposure [61]. Cui et al. [61] investigated the physiological changes of *Leymus secalinus* and the effect of altitude, observing an increase in soluble sugars as elevation increased but a decrease in chlorophyll *a* and *b*, leading to a decrease in the crop’s ability to absorb light. Both these reactions were noted as defence mechanisms and adaption strategies to the change in environment. It is possible that these environmental differences led the Spanish celery to synthesise ketones and aldehydes in response to these abiotic stresses. The solar radiation would be significantly higher in the UK-grown celery due to the lower altitude along with growing in the summer months. This will increase the duration of light exposed to the crop and, thus, increase the rate of photosynthesis. Although not discussed in celery, higher exposure to UV-B in tree foliage led to an increase in flavonoids as a protective mechanism [62], and it is possible that a similar mechanism occurred in UK celery but for monoterpene production.

Synthesising aromatic compounds is a typical response from the crop to abiotic and biotic stresses for protection and adaption to the growing environment, and it is clear the celery grown in the UK reacted differently to the celery grown in Spain. Turner et al. [10] previously suggested that increased sesquiterpene and phthalide content was due to temperature stress, yet similar temperatures and other climate conditions were experienced by the Spanish crop, leading to variation in the chemical composition. Differences in soil, water and fertilizer composition used upon the UK- and Spanish-grown celery caused a change in the availability of minerals and elements to be used for primary and secondary metabolite production and, along with the placement of the field which altered the duration of light, caused a change in the crop’s defence mechanism and adaption strategy.

## 4. Conclusions

Geographical location displayed a strong influence over the aroma composition of eight celery genotypes, and the influence expressed by genotype remained significant. Changes in composition caused by these factors led to differences in the aroma profile and, hence, sensory differences between genotypes and celery grown in different geographical locations were identified. Completing volatile analysis and sensory evaluation of the eight genotypes of celery demonstrated that celery genotypes grown and harvested in the UK were perceived with a strong green aroma and cucumber flavour compared to the celery grown and harvested in Spain. A wider range of compound families were identified within Spanish celery samples, imparting a significantly different aroma profile, which was perceived to be more closely associated with fresh fennel and coriander flavour. Identifying more compounds, including aldehydes and ketones in Spanish-grown celery, allowed for the explanation of the association to cucumber flavour.

Combining findings presented in this study and in the previous study completed by the authors [10], the genetic make-up of the crop regulates the synthesis of primary and secondary metabolites in response to abiotic and biotic stresses. Nonetheless, the environmental stresses experienced by the UK and Spanish crops were different and, thus, a different defence mechanism was required. This was reflected by the number of compounds expressing significant differences between genotypes and the variation caused by genotype in the UK crop, as well as the variation in perception between genotypes from sensory evaluation. The influence of geographical location on the aroma composition was also evident through the variation observed due to the location, in addition to most compounds also expressing significant differences caused by geographical location. The chemical composition was different in both locations, mostly caused by the aldehyde and ketone contents that were expressed in a significantly higher proportion of the volatile composition when sampling celery grown in Spain. A similar response was observed between harvest years, whereby significant compositional differences when the warmer temperatures of 2018 celery were observed, ultimately leading to an increased sesquiterpene and phthalide content in the eight genotypes when grown in a considerably warmer climate in response to stress.

All eight genotypes used within these studies were observed to be influenced by both genotype and external factors, including the environment (air temperatures, soil temperatures, relative humidity), geographical location (altitude and placement of field) and agronomic techniques (application of fertilisers, water availability and irrigation systems). Two genotypes (12 and 25) demonstrated consistency in their performance across harvest year and location; 12 remained a high “extreme”, profiled with strong fresh coriander and fennel attribute notes, which was reflected through its abundance in strong aroma compounds. On the other hand, genotype 25 was presented as a low “extreme” and was only profiled with a cucumber flavour, expressing significant correlations with related compounds, predominantly, aldehydes and ketones. This consistency makes these lines strong candidates to drive breeding programmes aimed at developing celery with distinct flavour profiles that will appeal to different consumer groups.

With apparent differences in the aroma and sensory profile, identifying which harvest year, environment, geographical location and agronomy produced the most appealing celery is impossible to accomplish without carrying out consumer preference trials combined with sensory profiling. Combining the data collected from this study and experiences alike with consumer preference tests would aid in the identification of attributes that consumers find important in celery products, including preferences for sweet, bitter and flavour intensity. The findings from this study could be offered to celery breeders and fresh produce growers to guide celery production with aroma profile targets in mind. Furthermore, by educating breeders about the environment, including location, genotype and agronomy, a deeper understanding will be provided on the role these factors play in determining and influencing the aroma profile and, therefore, the sensory perception of celery. Combining all these considerations will lead to a higher quality and better tasting product. Additionally, selecting cultivars according to the growing environment rather than using the same cultivar across circumstances will allow for a more consistent product.

## Figures and Tables

**Table 1:** Percentage composition of volatile compounds identified in the headspace of eight celery genotypes using SPME GC/MS and harvested in UK 2018 and Spain 2019.

Code	Compound	LRI_exp_^A^	ID ^B^	Percentage Composition (%) ^C^																*p*-Value ^D^		
				UK								Spain										
				5	8	10	12	15	18	22	25	5	8	10	12	15	18	22	25	G ^E^	E ^F^	GxE ^G^
	Alcohols																					
A1	3-methyl-3-buten-1-ol	730	A	0.42 ± 0.08 ^abc^	0.31 ± 0.04 ^ab^	0.94 ± 0.27 ^c^	0.35 ± 0.14 ^abc^	0.22 ± 0.07 ^a^	0.23 ± 0.06 ^a^	0.30 ± 0.12 ^ab^	0.39 ± 0.06 ^abc^	0.60 ± 0.35 ^abc^	0.40 ± 0.06 ^ahc^	0.91 ± 0.27 ^bc^	0.59 ± 0.13 ^abc^	0.36 ± 0.05 ^abc^	0.57 ± 0.22 ^abc^	0.54 ± 0.02 ^abc^	0.49 ± 0.13 ^abc^	**	**	**
A2	2-methyl-1-butanol	742	A	nd ^a^	nd ^a^	nd ^a^	nd ^a^	nd ^a^	nd ^a^	nd ^a^	nd ^a^	0.10 ± 0.01 ^ab^	0.10 ± 0.03 ^ab^	0.12 ± 0.02^b^	0.11 ± 0.01 ^ab^	nd ^a^	0.10 ± 0.04 ^ab^	0.10 ± 0.05 ^ab^	0.10 ± 0.02 ^ab^	***	***	***
A3	(*E*)-2-penten-1-ol	758	A	0.73 ± 0.28 ^ab^	0.42 ± 0.16 ^ab^	0.64 ± 0.04 ^ab^	0.23 ± 0.08 ^a^	0.32 ± 0.09 ^ab^	0.65 ± 0.23 ^ab^	1.2 ± 0.54 ^ab^	0.50 ± 0.22 ^ab^	0.72 ± 0.34 ^ab^	1.3 ± 0.25^b^	1.1 ± 0.18 ^ab^	0.71 ± 0.09 ^ab^	0.60 ± 0.09 ^ab^	0.81 ± 0.31 ^ab^	0.87 ± 0.24 ^ab^	0.52 ± 0.06 ^ab^	**	*	*
A4	1-pentanol	763	A	0.21 ± 0.06 ^a^	0.11 ± 0.04 ^a^	0.31 ± 0.20 ^a^	0.13 ± 0.10 ^a^	0.23 ± 0.15 ^a^	0.39 ± 0.14 ^ab^	0.63 ± 0.25 ^ab^	0.28 ± 0.08 ^a^	1.6 ± 0.27^b^	0.50 ± 0.11 ^a^	0.76 ± 0.28 ^ab^	0.49 ± 0.06 ^a^	1.1 ± 0.13 ^ab^	0.87 ± 0.34 ^ab^	1.5 ± 0.51^b^	0.88 ± 0.22 ^ab^	***	***	***
A5	1-hexanol	862	A	nd ^a^	nd ^a^	nd ^a^	nd ^a^	nd ^a^	nd ^a^	nd ^a^	nd ^a^	0.53 ± 0.19 ^ab^	0.44 ± 0.27 ^ab^	0.79 ± 0.44 ^b^	0.40 ± 0.21 ^ab^	0.33 ± 0.08 ^ab^	0.40 ± 0.10 ^ab^	0.48 ± 0.14 ^ab^	0.47 ± 0.23 ^ab^	***	***	***
	Total			1.4	0.84	1.9	0.71	0.77	1.3	2.1	1.2	3.5	2.7	3.7	2.3	2.4	2.7	3.5	2.5			
	Aldehydes																					
AH1	2-methyl-2-butenal	739	A	nd ^a^	nd ^a^	nd ^a^	nd ^a^	nd ^a^	nd ^a^	nd ^a^	nd ^a^	0.16 ± 0.07 ^bc^	0.15 ± 0.08 ^bc^	0.14 ± 0.06 ^bc^	0.13 ± 0.02 ^abc^	0.23 ± 0.03 ^c^	0.19 ± 0.04^b c^	0.19 ± 0.05 ^bc^	0.10 ± 0.03 ^ab^	***	***	***
AH2	(*E*)-2-pentenal	753	A	nd ^a^	nd ^a^	nd ^a^	nd ^a^	nd ^a^	nd ^a^	nd ^a^	nd ^a^	0.78 ± 0.04 ^c^	0.13 ± 0.08 ^a^	0.34 ± 0.14 ^ab^	nd ^a^	0.78 ± 0.08 ^c^	0.80 ± 0.36 ^c^	0.77 ± 0.09 ^bc^	0.38 ± 0.11 ^abc^	***	***	***
AH3	hexanal	800	A	9.7 ± 0.8 ^a^	1.3 ± 0.46 ^a^	2.6 ± 0.32 ^a^	0.65 ± 0.29 ^a^	2.0 ± 0.39 ^a^	8.9 ± 2.7 ^a^	13 ± 5.5 ^a^	6.3 ± 1.2 ^a^	25 ± 7.8 ^a^	24 ± 6.2 ^a^	14 ± 5.2 ^a^	8.6 ± 3.6 ^a^	22 ± 7.5 ^a^	24 ± 4.9 ^a^	25 ± 7.0 ^a^	22 ± 6.3 ^a^	**	**	**
AH4	(*E*)-2-hexenal	849	A	0.18 ± 0.11 ^abc^	tr ± 0.02 ^a^	tr ± 0.02 ^a^	0.04 ± 0.01 ^ab^	0.03 ± 0.03 ^a^	0.15 ± 0.11 ^abc^	0.20 ± 0.08 ^abc^	0.11 ± 0.05 ^abc^	0.56 ± 0.13 ^c^	0.57 ± 0.24^c^	0.30 ± 0.10 ^abc^	0.30 ± 0.07 ^abc^	0.55 ± 0.11 ^c^	0.54 ± 0.19 ^c^	0.57 ± 0.15 ^c^	0.51 ± 0.20 ^bc^	***	***	***
AH5	heptanal	901	A	tr ± 0.03 ^ab^	nd ^a^	0.28 ± 0.15 ^ab^	0.16 ± 0.13 ^ab^	0.25 ± 0.16 ^ab^	0.23 ± 0.14 ^ab^	0.29 ± 0.08 ^ab^	0.25 ± 0.15 ^ab^	0.68 ± 0.18 ^b^	0.58 ± 0.18 ^ab^	0.51 ± 0.13 ^ab^	0.48 ± 0.10 ^ab^	0.49 ± 0.35 ^ab^	0.57 ± 0.13 ^ab^	0.61 ± 0.20 ^ab^	0.72 ± 0.12^b^	**	**	**
AH6	(*E*)-2-heptenal	954	A	0.19 ± 0.22 ^a^	1.6 ± 0.55 ^ab^	1.6 ± 0.23 ^ab^	0.52 ± 0.04 ^a^	1.5 ± 0.10 ^ab^	3.2 ± 1.5 ^abc^	4.2 ± 1.3 ^abc^	1.8 ± 0.97 ^ab^	6.4 ± 0.75 ^bcd^	8.1 ± 0.23 ^cd^	6.0 ± 0.36 ^bcd^	6.1 ± 0.64 ^bcd^	11 ± 0.55 ^d^	7.8 ± 0.33 ^cd^	7.3 ± 0.45 ^cd^	7.5 ± 0.40 ^cd^	***	***	***
AH7	benzaldehyde	969	A	nd ^a^	nd ^a^	nd ^a^	nd ^a^	nd ^a^	nd ^a^	nd ^a^	nd ^a^	3.3 ± 1.8 ^b^	1.7 ± 0.50 ^ab^	1.9 ± 0.14 ^b^	1.9 ± 0.26 ^b^	1.7 ± 0.10 ^ab^	1.6 ± 0.48 ^ab^	1.7 ± 0.22 ^ab^	1.9 ± 0.22 ^b^	***	***	***
AH8	*n*-octanal	1007	A	0.10 ± 0.10 ^ab^	nd ^a^	0.49 ± 0.06 ^abcd^	0.27 ± 0.06 ^abc^	0.39 ± 0.19 ^abcd^	0.51 ± 0.26 ^abcd^	0.51 ± 0.17 ^abcd^	0.51 ± 0.23 ^abcd^	0.86 ± 0.19 ^cd^	0.95 ± 0.22 ^cde^	0.56 ± 0.10 ^abcd^	0.63 ± 0.13 ^abcd^	1.6 ± 0.35 ^e^	0.78 ± 0.21 ^bcd^	0.54 ± 0.04 ^abcd^	1.0 ± 0.22 ^de^	***	***	***
AH9	phenacetaldehyde	1049	A	nd ^a^	nd ^a^	nd ^a^	nd ^a^	nd ^a^	nd ^a^	nd ^a^	nd ^a^	0.31 ± 0.13 ^bc^	0.24 ± 0.04 ^bc^	0.26 ± 0.06 ^bc^	0.42 ± 0.06 ^c^	0.26 ± 0.02 ^bc^	0.24 ± 0.06 ^bc^	0.23 ± 0.98^b^	0.29 ± 0.05 ^bc^	***	***	***
AH10	(*E*)-2-octenal	1057	A	nd ^a^	nd ^a^	nd ^a^	nd ^a^	nd ^a^	nd ^a^	nd ^a^	nd ^a^	3.3 ± 1.3 ^b^	2.2 ± 1.5 ^ab^	1.5 ± 0.39 ^ab^	1.4 ± 0.39 ^ab^	3.4 ± 0.89 ^b^	3.5 ± 1.2 ^b^	2.8 ± 0.96 ^b^	3.5 ± 1.0 ^b^	***	***	***
AH11	*m*-tolualdehyde	1086	B [18]	0.33 ± 0.07 ^a^	0.24 ± 0.02 ^a^	4.0 ± 0.28 ^c^	1.1 ± 0.28 ^ab^	0.95 ± 0.02 ^ab^	0.19 ± 0.02 ^a^	0.26 ± 0.05 ^a^	1.6 ± 0.29 ^b^	0.72 ± 0.57 ^ab^	0.66 ± 0.26 ^ab^	0.71 ± 0.17 ^ab^	0.91 ± 0.19 ^ab^	0.64 ± 0.06 ^ab^	0.68 ± 0.32 ^ab^	0.57 ± 0.10 ^a^	0.97 ± 0.08 ^ab^	***	***	***
AH12	nonanal	1105	A	0.33 ± 0.14 ^abc^	0.12 ± 0.02 ^ab^	0.20 ± 0.03 ^abc^	0.10 ± 0.01 ^a^	0.17 ± 0.03 ^abc^	0.16 ± 0.10 ^abc^	0.22 ± 0.17 ^abc^	0.19 ± 0.09 ^abc^	0.68 ± 0.11 ^c^	0.59 ± 0.18 ^abc^	0.39 ± 0.10 ^b^	0.35 ± 0.13 ^abc^	0.57 ± 0.16 ^abc^	0.64 ± 0.35 ^bc^	0.61 ± 0.08 ^abc^	0.59 ± 0.11 ^abc^	***	***	***
AH13	(*E*,*E*)-2,4-octadienal	1110	A	nd ^a^	nd ^a^	nd ^a^	nd ^a^	nd ^a^	nd ^a^	nd ^a^	nd ^a^	0.15 ± 0.05 ^b^	0.13 ± 0.04 ^b^	0.11 ± 0.01 ^b^	0.13 ± 0.03 ^b^	0.16 ± 0.02 ^b^	0.15 ± 0.03 ^b^	0.14 ± 0.05 ^b^	0.20 ± 0.02 ^b^	***	***	***
AH14	(*E*,*Z*)-2,6-nonadienal	1162	A	nd ^a^	nd ^a^	nd ^a^	nd ^a^	nd ^a^	nd ^a^	nd ^a^	nd ^a^	0.10 ± 0.06 ^ab^	0.15 ± 0.03 ^abc^	0.11 ± 0.02 ^abc^	0.12 ± 0.02 ^abc^	0.29 ± 0.10 ^c^	0.23 ± 0.02 ^bc^	0.23 ± 0.16 ^bc^	0.28 ± 0.05 ^c^	***	***	***
AH15	(*E*)-2-nonenal	1165	A	nd ^a^	nd ^a^	nd ^a^	nd ^a^	nd ^a^	nd ^a^	nd ^a^	nd ^a^	0.10 ± 0.03 ^ab^	0.10 ± 0.02 ^ab^	tr ± 0.03 ^ab^	0.14 ± 0.02 ^b^	0.10 ± 0.01 ^ab^	0.10 ± 0.01 ^ab^	tr ± 0.05 ^ab^	0.12 ± 0.10 ^b^	***	***	***
AH16	myrtenal	1207	B [19]	nd ^a^	nd ^a^	nd ^a^	nd ^a^	nd ^a^	nd ^a^	nd ^a^	nd ^a^	0.19 ± 0.02 ^ab^	0.14 ± 0.02 ^a^	0.10 ± 0.03 ^a^	0.11 ± 0.01 ^a^	0.16 ± 0.04 ^ab^	0.15 ± 0.04 ^ab^	0.10 ± 0.06 ^a^	0.37 ± 0.21 ^b^	***	***	***
AH17	(*E*,*E*)-2,6-nonadienal	1156	A	0.21 ± 0.04 ^ab^	0.30 ± 0.03 ^ab^	0.18 ± 0.02 ^ab^	0.18 ± 0.04 ^ab^	0.17 ± 0.03 ^ab^	0.16 ± 0.08 ^ab^	tr ± 0.03 ^a^	0.22 ± 0.08 ^ab^	0.36 ± 0.11 ^ab^	0.48 ± 0.24 ^b^	0.20 ± 0.03 ^ab^	0.16 ± 0.05 ^ab^	0.41 ± 0.11 ^ab^	0.35 ± 0.11 ^ab^	0.46 ± 0.22 ^ab^	0.20 ± 0.17 ^ab^	*	*	*
	Total			11	3.6	9.4	3.0	5.5	14	19	11	44	41	28	23	44	44	43	41			
	Esters																					
E1	methyl butanoate	717	A	tr ± 0.03 ^abc^	tr ± 0.01 ^a^	tr ± 0.02 ^abc^	tr ± 0.01 ^ab^	tr ± 0.02 ^ab^	tr ± 0.04 ^ab^	tr ± 0.05 ^ab^	tr ± 0.01 ^ab^	0.22 ± 0.14 ^cd^	0.18 ± 0.01 ^abcd^	0.25 ± 0.04 ^d^	0.17 ± 0.01 ^abcd^	0.18 ± 0.04 ^abcd^	0.18 ± 0.04 ^abcd^	0.16 ± 0.02 ^abcd^	0.19 ± 0.03 ^bcd^	***	***	***
E2	methyl pentanoate	837	A	nd ^a^	nd ^a^	nd ^a^	nd ^a^	nd ^a^	nd ^a^	nd ^a^	nd ^a^	0.34 ± 0.23 ^b^	0.24 ± 0.02 ^ab^	0.37 ± 0.13 ^b^	0.40 ± 0.09 ^b^	0.23 ± 0.07 ^ab^	0.39 ± 0.18 ^b^	0.27 ± 0.05 ^ab^	0.30 ± 0.05 ^ab^	***	***	***
E3	methyl hexanoate	921	A	nd ^a^	nd ^a^	nd ^a^	nd ^a^	nd ^a^	nd ^a^	nd ^a^	nd ^a^	0.25 ± 0.12 ^ab^	0.29 ± 0.16 ^ab^	0.12 ± 0.01 ^ab^	0.10 ± 0.03 ^ab^	0.25 ± 0.09 ^ab^	0.38 ± 0.10 ^b^	0.28 ± 0.10 ^bc^	0.24 ± 0.11 ^ab^	***	***	***
E4	carveol acetate	1343	B [20]	nd ^a^	nd ^a^	nd ^a^	nd ^a^	nd ^a^	nd ^a^	nd ^a^	nd ^a^	0.21 ± 0.05 ^bc^	0.14 ± 0.02 ^ab^	0.22 ± 0.04 ^bc^	0.17 ± 0.04 ^bc^	0.20 ± 0.04 ^bc^	0.27 ± 0.08 ^bc^	0.20 ± 0.05 ^a^	0.29 ± 0.10 ^c^	***	***	***
E5	hexyl isobutanoate	1378	B [21]	0.10 ± 0.03	0.10 ± 0.04	0.14 ± 0.02	tr ± 0.03	0.10 ± 0.05	0.16 ± 0.04	0.32 ± 0.06	0.12 ± 0.03	0.15 ± 0.12	0.15 ± 0.12	0.40 ± 0.04	0.22 ± 0.11	0.18 ± 0.13	0.11 ± 0.16	0.36 ± 0.23	0.13 ± 0.11	ns	ns	ns
	Total			0.14	0.10	0.20	0.07	0.11	0.19	0.36	0.14	1.2	1.0	1.4	1.0	1.0	1.3	1.3	1.2			
	Ketones																					
K1	2-methyl-3-pentanone	746	A	nd ^a^	nd ^a^	nd ^a^	nd ^a^	nd ^a^	nd ^a^	nd ^a^	nd ^a^	0.10 ± 0.05 ^ab^	0.10 ± 0.02 ^ab^	0.19 ± 0.02 ^b^	0.10 ± 0.01 ^ab^	0.10 ± 0.01 ^a^	0.10 ± 0.02 ^ab^	0.10 ± 0.01 ^ab^	0.10 ± 0.02 ^ab^	***	***	***
K2	3-heptanone	884	A	nd ^a^	nd ^a^	nd ^a^	nd ^a^	nd ^a^	nd ^a^	nd ^a^	nd ^a^	0.14 ± 0.05 ^a^	0.13 ± 0.08 ^a^	0.12 ± 0.08 ^a^	tr ± 0.02 ^a^	0.10 ± 0.03 ^a^	0.13 ± 0.01 ^a^	0.13 ± 0.03 ^a^	0.13 ± 0.04 ^a^	***	***	**
K3	2-heptanone	889	A	nd ^a^	nd ^a^	nd ^a^	nd ^a^	nd ^a^	nd ^a^	nd ^a^	nd ^a^	0.49 ± 0.14 ^b^	0.48 ± 0.15 ^b^	0.31 ± 0.08 ^ab^	0.17 ± 0.12 ^ab^	0.39 ± 0.08 ^ab^	0.49 ± 0.12 ^b^	0.44 ± 0.16 ^b^	0.56 ± 0.18 ^b^	***	***	**
K4	1-octen-3-one	976	A	nd ^a^	nd ^a^	nd ^a^	nd ^a^	nd ^a^	nd ^a^	nd ^a^	nd ^a^	3.0 ± 0.55 ^b^	3.9 ± 1.7 ^b^	2.9 ± 0.17 ^b^	2.3 ± 0.35 ^ab^	4.4 ± 0.61 ^b^	3.3 ± 0.73 ^b^	3.5 ± 1.3 ^b^	3.9 ± 0.95 ^b^	***	***	**
K5	(*E*,*E*)-3,5-octadien-2-one	1070	B [22]	nd ^a^	nd ^a^	nd ^a^	nd ^a^	nd ^a^	nd ^a^	nd ^a^	nd ^a^	0.79 ± 0.14 ^b^	1.1 ± 0.29 ^b^	0.60 ± 0.14 ^ab^	0.81 ± 0.23 ^b^	1.3 ± 0.15 ^b^	0.82 ± 0.19 ^b^	1.3 ± 0.41 ^b^	0.63 ± 0.45 ^ab^	***	***	***
K6	acetophenone	1073	A	nd ^a^	nd ^a^	nd ^a^	nd ^a^	nd ^a^	nd ^a^	nd ^a^	nd ^a^	0.30 ± 0.16 ^b^	0.25 ± 0.16 ^b^	0.27 ± 0.05 ^b^	0.31 ± 0.04 ^b^	0.25 ± 0.01 ^b^	0.26 ± 0.07 ^b^	0.28 ± 0.07 ^b^	0.29 ± 0.02 ^b^	***	***	***
K7	3,5-octadien-2-one	1092	A	nd ^a^	nd ^a^	nd ^a^	nd ^a^	nd ^a^	nd ^a^	nd ^a^	nd ^a^	2.2 ± 0.65 ^b^	2.4 ± 1.1 ^b^	0.92 ± 0.38 ^ab^	0.81 ± 0.32 ^ab^	2.1 ± 0.77 ^b^	2.2 ± 1.0 ^b^	2.2 ± 0.81 ^b^	2.1 ± 0.91 ^ab^	***	***	***
K8	*p*-methyl-acetophenone	1179	B [23]	nd ^a^	nd ^a^	nd ^a^	nd ^a^	nd ^a^	nd ^a^	nd ^a^	nd ^a^	0.11 ± 0.04 ^ab^	0.10 ± 0.01 ^a^	tr ± 0.03 ^a^	0.10 ± 0.04 ^a^	0.10 ± 0.04 ^ab^	nd ^a^	0.10 ± 0.05	0.22 ± 0.10 ^b^	***	***	*
K9	dihydrojasmone	1378	A	nd ^a^	nd ^a^	nd ^a^	nd ^a^	nd ^a^	nd ^a^	nd ^a^	nd ^a^	0.62 ± 0.33 ^ab^	0.69 ± 0.38 ^b^	0.06 ± 0.04 ^ab^	0.17 ± 0.13 ^ab^	0.71 ± 0.36 ^b^	0.63 ± 0.26 ^ab^	0.30 ± 0.21 ^ab^	0.57 ± 0.15 ^ab^	***	***	***
	Total			0	0	0	0	0	0	0	0	7.8	9.1	5.4	4.8	9.4	7.9	8.3	8.5			
	Alkanes																					
ALK1	nonane	900	A	0.41 ± 0.15 ^ab^	0.32 ± 0.11 ^ab^	0.43 ± 0.19 ^ab^	0.14 ± 0.18 ^a^	0.13 ± 0.10 ^a^	0.28 ± 0.11 ^ab^	nd ^a^	0.17 ± 0.02 ^a^	0.84 ± 0.44 ^ab^	0.62 ± 0.36 ^ab^	0.69 ± 0.21 ^ab^	0.27 ± 0.14 ^a^	1.7 ± 0.34 ^b^	0.41 ± 0.06 ^ab^	0.36 ± 0.16 ^ab^	0.90 ± 0.35 ^ab^	*	*	*
ALK2	decane	1000	A	0.80 ± 0.24 ^abcd^	0.49 ± 0.13 ^ab^	nd ^a^	0.37 ± 0.11 ^ab^	0.60 ± 0.26 ^abc^	1.1 ± 0.21 ^bcde^	1.7 ± 0.29 ^ef^	0.83 ± 0.33 ^abcd^	1.6 ± 0.18 ^def^	1.7 ± 0.33 ^ef^	1.5 ± 0.36 ^cdef^	1.6 ± 0.05 ^def^	2.2 ± 0.21 ^f^	1.9 ± 0.05 ^ef^	1.9 ± 0.18 ^ef^	1.6 ± 0.19 ^def^	***	***	***
ALK3	undecane	1100	A	0.26 ± 0.15 ^abcd^	0.14 ± 0.09	0.19 ± 0.11 ^abcd^	0.04 ± 0.05 ^a^	0.24 ± 0.06 ^abc^	0.14 ± 0.10 ^abc^	0.07 ± 0.08 ^a^	0.11 ± 0.06 ^ab^	0.60 ± 0.31 ^cd^	0.27 ± 0.10 ^abcd^	0.57 ± 0.04 ^bcd^	0.63 ± 0.02 ^f^	0.55 ± 0.03 ^bcd^	0.33 ± 0.03 ^abcd^	0.43 ± 0.12 ^abcd^	0.52 ± 0.05 ^abcd^	***	***	***
ALK4	dodecane	1199	A	0.48 ± 0.08	0.37 ± 0.03	0.46 ± 0.05	0.31 ± 0.10	0.33 ± 0.10	0.44 ± 0.13	0.46 ± 0.10	0.44 ± 0.12	0.48 ± 0.23	0.20 ± 0.03	0.37 ± 0.10	0.31 ± 0.05	0.26 ± 0.03	0.29 ± 0.03	0.27 ± 0.04	0.34 ± 0.08	ns	ns	ns
ALK5	tridecane	1299	A	nd	nd	nd	nd	nd	nd	nd	nd	0.16 ± 0.03	nd	nd	nd	nd	nd	nd	nd	ns	ns	ns
ALK6	tetradecane	1399	A	0.11 ± 0.02	tr ± 0.03	tr ± 0.02	tr ± 0.03	0.10 ± 0.06	0.10 ± 0.03	tr ± 0.03	0.10 ± 0.02	0.16 ± 0.12	tr ± 0.03	tr ± 0.01	tr ± 0.01	tr ± 0.01	tr ± 0.03	tr ± 0.02	0.10 ± 0.06	ns	ns	ns
ALK7	pentadecane	1499	A	nd ^a^	nd ^a^	nd ^a^	nd ^a^	nd ^a^	nd ^a^	nd ^a^	nd ^a^	0.15 ± 0.02 ^a^	nd ^a^	tr ± 0.05 ^a^	nd ^a^	0.18 ± 0.02 ^a^	0.14 ± 0.01 ^a^	0.14 ± 0.02 ^a^	nd ^a^	**	**	**
	Total			2.1	1.4	1.1	0.94	1.4	2.1	2.3	1.6	4.0	2.8	3.2	2.8	4.9	3.1	3.1	3.4			
	Monoterpenes																					
M1	α-thujene	933	B [24]	0.27 ± 0.09	0.24 ± 0.08	0.29 ± 0.13	0.30 ± 0.11	0.22 ± 0.10	0.41 ± 0.19	0.32 ± 0.14	0.22 ± 0.13	0.64 ± 0.31	0.52 ± 0.19	1.1 ± 0.17	0.78 ± 0.20	0.42 ± 0.02	0.58 ± 0.14	0.64 ± 0.06	0.72 ± 0.22	ns	ns	ns
M2	α-pinene	943	A	0.62 ± 0.05	0.85 ± 0.22	0.52 ± 0.19	0.62 ± 0.18	1.0 ± 0.42	0.89 ± 0.20	0.43 ± 0.20	0.62 ± 0.31	0.83 ± 0.14	0.49 ± 0.26	1.0 ± 0.30	0.81 ± 0.16	0.77 ± 0.33	0.69 ± 0.10	1.1 ± 0.58	0.75 ± 0.46	ns	ns	ns
M3	camphene	960	A	2.5 ± 0.5	0.33 ± 0.07	0.29 ± 0.12	0.21 ± 0.08	0.35 ± 0.10	0.48 ± 0.05	0.66 ± 0.26	0.22 ± 0.08	0.73 ± 0.21	0.57 ± 0.05	0.93 ± 0.05	0.94 ± 0.13	0.73 ± 0.12	0.45 ± 0.32	0.96 ± 0.11	0.68 ± 0.14	ns	ns	ns
M4	sabinene	981	A	0.44 ± 0.13	0.33 ± 0.04	0.66 ± 0.39	0.27 ± 0.04	0.28 ± 0.05	0.45 ± 0.03	0.53 ± 0.13	0.36 ± 0.06	0.37 ± 0.25	0.29 ± 0.08	0.34 ± 0.19	0.32 ± 0.09	0.31 ± 0.08	0.38 ± 0.15	0.30 ± 0.07	0.34 ± 0.07	ns	ns	ns
M5	β-pinene	989	A	3.0 ± 0.64	5.2 ± 1.6	0.96 ± 0.36	5.4 ± 1.6	3.8 ± 1.6	2.7 ± 0.99	0.79 ± 0.24	4.5 ± 1.1	2.3 ± 0.63	2.1 ± 1.1	1.5 ± 0.38	2.6 ± 0.65	3.5 ± 1.4	1.1 ± 0.18	2.5 ± 1.3	2.9 ± 1.9	ns	ns	ns
M6	myrcene	992	A	1.1 ± 0.26 ^abc^	1.9 ± 0.64 ^abc^	2.6 ± 0.74^bc^	2.6 ± 0.22^bc^	1.6 ± 0.37 ^abc^	2.1 ± 0.61 ^abc^	0.84± 0.34 ^ab^	1.1 ± 0.45 ^abc^	0.51 ± 0.03 ^a^	0.54± 0.19 ^ab^	1.8 ± 0.46 ^abc^	1.4 ± 0.06 ^abc^	0.48 ± 0.10 ^a^	1.1 ± 0.25 ^abc^	0.56 ± 0.18 ^ab^	0.51 ± 0.05 ^a^	***	***	***
M7	α-phellandrene	1013	A	nd ^a^	nd ^a^	nd ^a^	nd ^a^	nd ^a^	nd ^a^	nd ^a^	nd ^a^	0.37 ± 0.16 ^bc^	0.31 ± 0.03 ^b^	0.52 ± 0.06 ^c^	0.40 ± 0.06 ^bc^	0.33 ± 0.04 ^b^	0.39 ± 0.03 ^bc^	0.39 ± 0.07 ^bc^	0.37 ± 0.03 ^bc^	***	***	***
M8	Δ -3-carene	1019	A	0.24 ± 0.10	0.23 ± 0.18	0.25 ± 0.04	0.25 ± 0.12	0.22 ± 0.11	0.21 ± 0.10	0.32 ± 0.09	0.23 ± 0.05	0.72 ± 0.33	0.69 ± 0.39	0.94 ± 0.74	0.63 ± 0.44	0.54 ± 0.30	0.58 ± 0.30	0.77 ± 0.38	0.77 ± 0.46	ns	ns	ns
M9	*m*-cymene	1032	A	4.3 ± 0.61	3.6 ± 0.41	3.5 ± 0.69	3.8 ± 0.43	3.4 ± 0.78 ^a^	5.0 ± 0.71	2.8 ± 0.61	3.7 ± 0.55	3.8 ± 0.94	3.7 ± 1.1	4.6 ± 1.3	3.4 ± 0.67	2.3 ± 0.94	3.9 ± 0.82	3.4 ± 1.5	3.3 ± 1.1	ns	ns	ns
M10	limonene	1034	A	39 ± 8.2^bc^	43 ± 0.56^c^	33 ± 5.1 ^abc^	32 ± 2.3 ^abc^	39 ± 3.1^bc^	32 ± 4.5 ^abc^	29 ± 3.9 ^abc^	33 ± 3.1 ^abc^	11 ± 4.9 ^a^	19 ± 1.9 ^abc^	24 ± 7.6 ^abc^	21 ± 2.1 ^abc^	11 ± 6.1 ^a^	12 ± 5.1 ^a^	15 ± 5.3 ^ab^	11 ± 5.3 ^a^	***	***	***
M11	β-(*E*)-ocimene	1049	B [25]	0.19 ± 0.01 ^a^	0.18 ± 0.07 ^a^	0.17 ± 0.02 ^a^	0.24 ± 0.03 ^a^	0.17 ± 0.02 ^a^	0.16 ± 0.02 ^a^	0.42 ± 0.08 ^a^	0.18 ± 0.02 ^a^	1.3 ± 0.91 ^ab^	0.71 ± 0.32 ^a^	nd ^a^	nd ^a^	1.7 ± 0.29 ^ab^	1.1 ± 0.28 ^a^	nd ^a^	3.1 ± 0.43 ^b^	***	***	***
M12	γ-terpinene	1066	A	4.2 ± 1.2^bcd^	4.3 ± 1.2 ^bcd^	3.6 ± 0.60 ^abcd^	5.9 ± 0.28 ^d^	5.6 ± 0.27 ^cd^	5.5 ± 1.4 ^cd^	2.1 ± 0.90 ^ab^	5.6 ± 1.4 ^d^	0.72 ± 0.12 ^a^	2.6 ± 1.4 ^abcd^	2.2 ± 0.36 ^abc^	2.0 ± 0.35 ^ab^	1.2 ± 0.24 ^ab^	1.1 ± 0.24 ^ab^	1.1 ± 0.20 ^ab^	1.1 ± 0.36 ^ab^	***	***	***
M13	terpinolene	1097	A	0.62 ± 0.19 ^abc^	0.89 ± 0.07 ^c^	0.53 ± 0.09 ^abc^	0.43 ± 0.01 ^abc^	0.36 ± 0.22 ^abc^	0.73 ± 0.20 ^bc^	0.57 ± 0.14 ^abc^	0.90 ± 0.31 ^c^	0.35 ± 0.08 ^abc^	0.25 ± 0.18 ^abc^	0.13 ± 0.08 ^ab^	0.20 ± 0.14 ^ab^	0.38 ± 0.14 ^abc^	0.34 ± 0.14 ^abc^	nd ^a^	0.25 ± 0.18 ^abc^	***	***	**
M14	*allo*-ocimene	1132	B [26]	0.11 ± 0.06 ^ab^	0.10 ± 0.01 ^ab^	0.10 ± 0.05 ^ab^	0.31 ± 0.03 ^b^	0.24 ± <0.01 ^ab^	0.13 ± 0.04 ^ab^	0.31 ± 0.27 ^b^	0.13 ± 0.08 ^ab^	nd ^a^	nd ^a^	nd ^a^	nd ^a^	nd ^a^	nd ^a^	nd ^a^	nd ^a^	***	***	**
M15	β-thujone	1124	B [23]	nd ^a^	nd ^a^	nd ^a^	nd ^a^	nd ^a^	nd ^a^	nd ^a^	nd ^a^	0.10 ± 0.02 ^ab^	tr ± 0.02 ^a^	0.10 ± 0.01 ^abc^	0.20 ± 0.04 ^c^	tr ± 0.02 ^ab^	0.10 ± 0.02 ^ab^	0.17 ± 0.12^bc^	0.10 ± 0.02 ^ab^	***	***	***
M16	*p*-mentha-1,5,8-triene	1135	B [27]	0.26 ± 0.05 ^ab^	0.10 ± 0.01 ^ab^	0.22 ± 0.02 ^ab^	0.56 ± 0.09 ^b^	0.26 ± 0.07 ^ab^	0.13 ± 0.09 ^ab^	0.49 ± 0.17 ^ab^	0.19 ± 0.08 ^ab^	0.10 ± 0.02 ^ab^	tr ± 0.02 ^a^	0.16 ± 0.04 ^ab^	0.55 ± 0.15 ^ab^	0.10 ± 0.01 ^ab^	0.17 ± 0.05 ^ab^	0.50 ± 0.27 ^ab^	0.10 ± 0.06 ^ab^	**	**	**
M17	(*Z*)-carveol	1147	B [19]	0.48 ± 0.13 ^bcd^	0.57 ± 0.17 ^cd^	0.23 ± 0.08 ^abc^	0.18 ± 0.08 ^ab^	0.24 ± 0.02 ^ab^	0.31 ± 0.21 ^abc^	tr ± 0.03 ^a^	0.13 ± 0.10 ^ab^	0.51 ± 0.07 ^cd^	0.45 ± 0.21 ^bcd^	0.65 ± 0.09^d^	0.44 ± 0.02 ^bcd^	0.34 ± 0.07 ^abcd^	0.51 ± 0.14 ^cd^	0.26 ± 0.09 ^abcd^	0.60 ± 0.23 ^d^	***	***	***
M18	pentylcyclohexa-1,3-diene	1166	B [19]	0.20 ± 0.05 ^ab^	0.23 ± 0.08 ^ab^	0.25 ± 0.03 ^ab^	0.46 ± 0.11 ^abc^	0.31 ± 0.03 ^ab^	0.10 ± 0.04 ^a^	0.26 ± 0.16 ^ab^	0.20 ± 0.01 ^ab^	0.20 ± 0.06 ^ab^	0.13 ± 0.09 ^a^	0.19 ± 0.08 ^ab^	0.20 ± 0.02 ^ab^	0.16 ± 0.05 ^ab^	0.19 ± 0.02 ^ab^	0.12 ± 0.09 ^a^	0.30 ± 0.14 ^ab^	*	*	*
M19	(*Z*)-dihydrocarvone	1208	A	0.39 ± 0.09 ^b^	0.36 ± 0.05 ^b^	0.35 ± 0.08 ^b^	0.19 ± 0.06 ^ab^	0.27 ± 0.05 ^ab^	0.18 ± 0.04 ^ab^	0.20 ± 0.08 ^ab^	0.26 ± 0.02 ^ab^	0.35 ± 0.03 ^b^	0.28 ± 0.02 ^ab^	0.30 ± 0.05 ^b^	0.25 ± 0.06 ^ab^	0.23 ± 0.12 ^ab^	0.20 ± 0.14 ^ab^	nd ^a^	0.39 ± 0.06 ^b^	**	**	**
M20	camphor	1157	A	nd ^a^	nd ^a^	nd ^a^	nd ^a^	nd ^a^	nd ^a^	nd ^a^	nd ^a^	0.27 ± 0.15 ^bc^	0.17 ± 0.04 ^abc^	0.22 ± 0.06 ^abc^	0.17 ± 0.05 ^abc^	0.18 ± 0.08 ^abc^	0.23 ± 0.06 ^bc^	0.15 ± 0.03 ^ab^	0.38 ± 0.13 ^c^	***	***	***
M21	isoborneol	1173	A	nd ^a^	nd ^a^	nd ^a^	nd ^a^	nd ^a^	nd ^a^	nd ^a^	nd ^a^	0.25 ± 0.14 ^b^	0.17 ± 0.03 ^ab^	0.16 ± 0.06 ^ab^	0.17 ± 0.04 ^ab^	0.19 ± 0.04 ^ab^	0.25 ± 0.04 ^b^	0.18 ± 0.05 ^ab^	0.23 ± 0.12 ^b^	***	***	***
M22	(*E*)-dihydrocarvone	1240	B [27]	0.79 ± 0.12 ^f^	0.79 ± 0.14 ^f^	0.67 ± 0.10 ^ef^	0.41 ± 0.08 ^cde^	0.57 ± 0.09 ^ef^	0.43 ± 0.05 ^de^	0.38 ± 0.06 ^bcde^	0.59 ± 0.03 ^ef^	0.10 ± 0.03 ^ab^	0.10 ± 0.04 ^a^	0.10 ± 0.02 ^ab^	0.10 ± 0.01 ^ab^	0.10 ± 0.02 ^a^	0.11 ± 0.03 ^abc^	tr ± 0.04 ^a^	0.14 ± 0.09 ^abcd^	***	***	***
M23	β-cyclocitral	1230	A	nd ^a^	nd ^a^	nd ^a^	nd ^a^	nd ^a^	nd ^a^	nd ^a^	nd ^a^	0.10 ± 0.04 ^b^	0.12 ± 0.02 ^b^	0.11 ± 0.03 ^b^	0.18 ± 0.02 ^b^	0.15 ± 0.01 ^b^	0.12 ± 0.02 ^b^	0.10 ± 0.01 ^b^	0.14 ± 0.06 ^b^	***	***	***
M24	L-carvone	1248	A	0.96 ± 0.19 ^bcd^	0.57 ± 0.11 ^abc^	1.5 ± 0.05 ^d^	0.71 ± 0.06 ^abc^	0.81 ± 0.13 ^abcd^	0.61 ± 0.14 ^abc^	0.75 ± 0.17 ^abcd^	1.1 ± 0.12 ^cd^	0.38 ± 0.22 ^abc^	0.26 ± 0.11 ^ab^	0.18 ± 0.06 ^ab^	0.14 ± 0.02 ^a^	0.23 ± 0.08 ^ab^	0.36 ± 0.03 ^abc^	0.17 ± 0.08 ^ab^	0.45 ± 0.23 ^abc^	***	***	***
M25	D-carvone	1262	A	0.43 ± 0.19	0.36 ± 0.10	0.24 ± 0.02	0.18 ± 0.03	0.23 ± 0.08	0.34 ± 0.15	0.44 ± 0.07	0.29 ± 0.06	0.33 ± 0.13	0.27 ± 0.06	0.60 ± 0.13	0.36 ± 0.17	0.30 ± 0.10	0.48 ± 0.11	0.52 ± 0.11	0.47 ± 0.18	ns	ns	ns
M26	thymol	1290	A	0.17 ± 0.05 ^b^	0.11 ± 0.14 ^ab^	0.12 ± 0.04 ^ab^	0.15 ± 0.09 ^ab^	0.11 ± 0.08 ^ab^	0.10 ± 0.03 ^ab^	nd ^a^	0.14 ± 0.11 ^ab^	0.15 ± 0.09 ^ab^	0.12 ± 0.07 ^ab^	0.15 ± 0.01 ^ab^	0.16 ± 0.01 ^ab^	0.12 ± 0.01 ^ab^	0.19 ± 0.08^b^	0.10 ± 0.03 ^ab^	0.16 ± 0.05 ^ab^	*	*	*
M27	carvacrol	1317	A	0.54 ± 0.08	0.42 ± 0.09	0.45 ± 0.03	0.60 ± 0.02	0.29 ± 0.03	0.39 ± 0.03	0.18 ± 0.04	0.52 ± 0.04	0.44 ± 0.21	0.36 ± 0.27	0.45 ± 0.05 ^a^	0.53 ± 0.08	0.31 ± 0.12	0.56 ± 0.23	0.19 ± 0.07	0.39 ± 0.14	ns	ns	ns
	Total			61	64	50	56	59	53	42	54	27	34	42	38	26	27	29	30			
	Monoterpenoid Alcohols																					
MA1	(+)-(*E*)-*p*-mentha-2,8-dien-1-ol	1122	A	0.10 ± 0.03	0.15 ± 0.01	tr ± 0.03	0.28 ± 0.03	0.10 ± 0.02	0.10 ± 0.03	tr ± 0.03	0.14 ± 0.01	0.15 ± 0.03	0.16 ± 0.01	0.15 ± 0.03	0.13 ± 0.02	0.12 ± 0.07	0.13 ± 0.02	0.12 ± 0.03	0.19 ± 0.13	ns	ns	ns
MA2	dihydrolinalool	1142	A	nd ^a^	nd ^a^	nd ^a^	nd ^a^	nd ^a^	nd ^a^	nd ^a^	nd ^a^	0.75 ± 0.31 ^abc^	0.33 ± 0.26 ^abc^	0.93 ± 0.08^bc^	1.2 ± 0.06^c^	0.78 ± 0.18 ^abc^	0.64 ± 0.30 ^abc^	0.29 ± 0.11 ^ab^	0.48 ± 0.24 ^abc^	***	***	***
MA3	(*Z*)-pinocarveol	1147	B [28]	0.59 ± 0.13 ^a^	0.63 ± 0.17 ^a^	0.30 ± 0.08 ^a^	0.20 ± 0.08 ^a^	0.28 ± 0.02 ^a^	0.35 ± 0.21 ^a^	tr ± 0.06 ^a^	0.45 ± 0.10 ^a^	0.29 ± 0.09 ^a^	0.21 ± 0.10 ^a^	0.11 ± 0.06 ^a^	0.10 ± 0.01 ^a^	0.20 ± 0.10 ^a^	0.47 ± 0.32 ^a^	0.15 ± 0.03 ^a^	0.57 ± 0.42 ^a^	*	*	*
MA4	terpinen-4-ol	1184	A	0.10 ± 0.01 ^ab^	nd ^a^	tr ± 0.03 ^a^	tr ± 0.03 ^ab^	tr ± 0.03 ^a^	0.10 ± 0.07 ^ab^	nd ^a^	0.13 ± 0.03 ^ab^	0.10 ± 0.09 ^ab^	0.15 ± 0.04 ^ab^	0.13 ± 0.03 ^ab^	0.18 ± 0.02 ^b^	0.10 ± 0.04 ^ab^	0.15 ± 0.06 ^ab^	nd ^a^	0.20 ± 0.04 ^b^	***	***	***
MA5	α-terpineol	1211	A	nd	nd	nd	nd	nd	nd	nd	nd	0.10 ± 0.04	nd	0.10 ± 0.01	0.10 ± 0.01	tr ± 0.03	0.10 ± 0.01	tr ± 0.03	0.13 ± 0.09	ns	ns	ns
MA6	(*E*)-8-hydroxylinalool	1349	B [19]	nd ^a^	nd ^a^	nd ^a^	nd ^a^	nd ^a^	nd ^a^	nd ^a^	nd ^a^	0.19 ± 0.05 ^b^	0.15 ± 0.06 ^b^	0.10 ± 0.04 ^ab^	0.10 ± 0.01 ^ab^	0.10 ± 0.02 ^ab^	0.18 ± 0.03 ^b^	0.10 ± 0.06 ^ab^	0.18 ± 0.05 ^b^	***	***	***
MA7	caryophylladienol II	1665	B [19]	nd ^a^	nd ^a^	nd ^a^	nd ^a^	nd ^a^	nd ^a^	nd ^a^	nd ^a^	0.1± 0.05 ^b^	nd ^a^	0.10± 0.01 ^b^	0.10± 0.02 ^b^	0.10± 0.01 ^b^	0.11± 0.03 ^b^	0.10 ± 0.02 ^b^	0.10 ± 0.03 ^b^	***	***	***
	Total			0.79	0.78	0.38	0.53	0.39	0.48	0.06	0.72	1.6	1.0	1.6	1.9	1.4	1.8	0.77	1.7			
	Sesquiterpenes																					
S1	α-ylangene	1384	B [27]	0.26 ± 0.11 ^c^	0.24 ± 0.07 ^c^	0.17 ± 0.11 ^c^	tr ± 0.01 ^ab^	0.16 ± 0.05 ^bc^	0.19 ± 0.10 ^c^	0.20 ± 0.26 ^c^	0.20 ± 0.14 ^c^	nd ^a^	nd ^a^	nd ^a^	nd ^a^	nd ^a^	nd ^a^	nd ^a^	nd ^a^	***	***	***
S2	α-copaene	1390	A	1.1 ± 0.02^e^	0.86 ± 0.01 ^de^	0.62 ± 0.03 ^cde^	0.10 ± 0.02 ^ab^	0.15 ± 0.05 ^ab^	0.49 ± 0.03 ^bcd^	0.78 ± 0.04 ^de^	0.77 ± 0.05 ^de^	0.14 ± 0.04 ^ab^	0.09 ± 0.06 ^ab^	0.06 ± 0.02 ^ab^	nd ^a^	nd ^a^	0.12 ± 0.05 ^ab^	0.24 ± 0.07 ^abc^	0.22 ± 0.18 ^abc^	***	***	***
S3	(*E*)-β-caryophyllene	1430	B [29]	tr ± 0.03	tr ± 0.02	nd	nd	tr ± 0.04	nd	nd	nd	nd	nd	nd	nd	nd	nd	nd	nd	ns	ns	ns
S4	β-caryophyllene	1445	A	4.4 ± 0.61 ^bc^	5.5 ± 0.32 ^c^	4.1 ± 0.43 ^bc^	2.5 ± 0.39 ^ab^	4.3 ± 1.3 ^bc^	4.1 ± 1.2 ^bc^	2.4 ± 0.29 ^ab^	2.2 ± 0.50 ^ab^	0.67 ± 0.52 ^a^	0.60 ± 0.40 ^a^	1.4 ± 0.73 ^a^	1.0 ± 0.15 ^a^	0.46 ± 0.17 ^a^	1.2 ± 0.13 ^a^	0.55 ± 0.28 ^a^	0.69 ± 0.28 ^a^	***	***	***
S5	(*+*)-aromadendrene	1452	A	0.17 ± 0.04 ^de^	0.21 ± 0.01 ^e^	0.15 ± 0.04 ^cde^	tr ± 0.07 ^abc^	0.13 ± 0.03 ^cde^	0.15 ± 0.08 ^cde^	0.10 ± 0.06 ^abc^	0.10 ± 0.01^bcd^	tr ± 0.01 ^ab^	nd ^a^	nd ^a^	nd ^a^	nd ^a^	nd ^a^	nd ^a^	nd ^a^	***	***	***
S6	curcumene	1472	B [30]	0.18 ± 0.09 ^abcd^	0.23 ± 0.11 ^b^	0.19 ± 0.06 ^b^	0.09 ± 0.05 ^a^	0.15 ± 0.22 ^b^	0.22 ± 0.19 ^b^	tr ± 0.03 ^bcde^	0.12 ± 0.05 ^a^	Nd ^a^	Nd ^a^	Nd ^a^	Nd ^a^	Nd ^a^	Nd ^a^	Nd ^a^	Nd ^a^	***	ns	***
S7	α-humulene	1479	A	0.42 ± 0.16 ^abcd^	0.70 ± 0.58 ^d^	0.38 ± 0.29 ^abcd^	0.49 ± 0.10 ^bcd^	0.51 ± 0.76 ^cd^	0.40 ± 0.65 ^abcd^	0.18 ± 0.01 ^abc^	0.26 ± 0.91 ^abcd^	0.11 ± 0.02 ^ab^	0.10 ± 0.06 ^a^	0.10 ± 0.05 ^a^	0.10 ± 0.02 ^a^	0.19 ± 0.04 ^abc^	0.10 ± 0.06 ^a^	tr ± 0.03 ^a^	0.13 ± 0.05 ^abc^	***	***	***
S8	β-selinene	1508	B [31]	3.0 ± 0.05 ^cd^	2.7 ± 0.06 ^bcd^	1.5 ± 0.02 ^abc^	4.6 ± 0.15 ^d^	2.2 ± 0.19 ^abcd^	1.9 ± 0.12 ^abc^	3.3 ± 0.26 ^cd^	3.0 ± 0.14 ^bcd^	0.35 ± 0.25 ^ab^	0.31 ± 0.16 ^ab^	0.31 ± 0.17 ^ab^	1.3 ± 0.29 ^abc^	0.17 ± 0.06 ^a^	0.40 ± 0.26 ^ab^	0.36 ± 0.15 ^ab^	0.50 ± 0.12 ^ab^	***	***	***
S9	valencene	1514	A	nd ^a^	nd ^a^	nd ^a^	2.9 ± 0.44 ^c^	nd ^a^	nd ^a^	nd ^a^	0.20 ± 0.07 ^a^	nd ^a^	nd ^a^	tr ± 0.02 ^a^	2.1 ± 0.16 ^b^	tr ± 0.02 ^a^	tr ± 0.01 ^a^	tr ± 0.02 ^a^	0.36 ± 0.05 ^a^	***	***	***
S10	α-selinene	1515	B [32]	0.61 ± 0.02 ^c^	0.60 ± 0.02 ^c^	0.43 ± 0.05 ^abc^	0.63 ± 0.44 ^c^	0.54 ± 0.04 ^bc^	0.44 ± 0.03 ^abc^	0.71 ± 0.02 ^c^	0.59 ± 0.07 ^c^	0.10 ± 0.04 ^a^	tr ± 0.03 ^a^	tr ± 0.03 ^a^	0.14 ± 0.03 ^ab^	tr ± 0.02 ^a^	tr ± 0.05 ^a^	tr ± 0.04 ^a^	0.10 ± 0.02 ^a^	***	***	***
S11	kessane	1557	B [19]	nd ^a^	0.12 ± 0.02 ^a^	nd ^a^	2.8 ± 0.05^c^	nd ^a^	nd ^a^	nd ^a^	nd ^a^	tr ± 0.03 ^a^	tr ± 0.01 ^a^	nd ^a^	2.0 ± 0.13^b^	nd ^a^	tr ± 0.02 ^a^	nd ^a^	0.36 ± 0.05 ^a^	***	***	***
S12	cuparene ^$^	1530	B [33]	nd	nd	nd	nd	nd	nd	nd	nd	tr ± 0.02	nd	nd	nd	tr ± 0.01	tr ± 0.01	nd	tr ± 0.04	ns	ns	ns
S13	(*E*)-nerolidol	1540	A	nd ^a^	nd ^a^	nd ^a^	nd ^a^	nd ^a^	nd ^a^	nd ^a^	nd ^a^	tr ± 0.02 ^a^	tr ± 0.02 ^a^	nd ^a^	nd ^a^	0.10 ± 0.02 ^a^	tr ± 0.04 ^a^	tr ± 0.03 ^a^	tr ± 0.03 ^a^	**	**	**
S14	liguloxide ^$^	1560	B [34]	nd ^a^	nd ^a^	nd ^a^	nd ^a^	nd ^a^	nd ^a^	nd ^a^	nd ^a^	nd ^a^	nd ^a^	nd ^a^	tr ± 0.01 ^a^	nd ^a^	tr ± 0.05 ^a^	nd ^a^	tr ± 0.01 ^a^	**	*	*
	Total			10	11	7.5	14	8.2	7.9	7.7	7.4	1.4	1.2	1.9	6.7	0.95	2.0	1.3	2.4			
	Phthalides																					
P1	3-butylhexahydro phthalide	1662	B [19]	nd ^a^	nd ^a^	nd ^a^	nd ^a^	nd ^a^	nd ^a^	nd ^a^	nd ^a^	tr ± 0.04 ^abc^	tr ± 0.02 ^ab^	tr ± 0.01 ^abc^	nd ^a^	0.10 ± 0.01^bc^	0.10 ± 0.02^c^	tr ± 0.01 ^abc^	0.10 ± 0.01^bc^	***	***	***
P2	3-n-butylphthalide	1676	B [8,10]	5.0 ± 0.01 ^abc^	5.2 ± 0.03 ^abc^	9.4 ± 0.05 ^cd^	6.6 ± 0.01 ^abcd^	7.1 ± 0.03 ^abcd^	6.7 ± 0.01 ^abcd^	9.8 ± 0.06 ^d^	7.0 ± 0.03 ^abcd^	4.2 ± 1.1 ^ab^	3.6 ± 0.81 ^a^	5.6 ± 1.1 ^abcd^	8.5 ± 0.86 ^bcd^	4.9 ± 0.93 ^ab^	5.6 ± 1.4 ^abcd^	5.2 ± 1.3 ^abc^	4.6 ± 0.87 ^ab^	***	***	***
P3	(*Z*)-3-butylidenephthalide	1685	B [19]	0.15 ± 0.06 ^ab^	0.22 ± 0.05 ^abc^	0.36 ± 0.09^b^	0.16 ± 0.02 ^ab^	0.25 ± 0.02 ^ab^	0.17 ± 0.07 ^ab^	0.25 ± 0.34 ^ab^	0.18 ± 0.25 ^ab^	0.22 ± 0.20 ^ab^	0.10 ± 0.04 ^a^	0.13 ± 0.01 ^ab^	0.13 ± 0.01 ^ab^	0.25 ± 0.06 ^ab^	0.17 ± 0.06 ^ab^	0.10 ± 0.01 ^a^	0.14 ± 0.04 ^ab^	*	*	*
P4	sedanenolide	1748	B [8,10]	4.8 ± 0.30 ^abcd^	9.7 ± 2.3 ^bcde^	15 ± 1.9 ^e^	16 ± 1.6 ^e^	14 ± 3.0 ^e^	9.5 ± 2.9 ^abcde^	11 ± 3.0 ^cde^	13 ± 2.2 ^de^	1.1 ± 0.30 ^ab^	0.96 ± 0.03 ^a^	3.7 ± 1.1 ^abc^	9.2 ± 1.1 ^abcde^	1.5 ± 0.49 ^ab^	2.0 ± 0.89 ^ab^	0.92 ± 0.52 ^a^	1.3 ± 1.1 ^ab^	***	***	***
P5	(*Z*)-neocnidilide	1755	B [19]	0.26 ± 0.03 ^a^	0.13 ± 0.03 ^a^	1.8 ± 0.02 ^c^	0.16 ± 0.04 ^a^	0.30 ± 0.06 ^ab^	0.78 ± 0.06 ^abc^	0.99 ± 0.04 ^abc^	0.94 ± 0.04 ^abc^	1.4 ± 1.1 ^abc^	0.45 ± 0.24 ^abc^	1.2 ± 0.24 ^abc^	0.14 ± 0.01 ^a^	0.37 ± 0.15 ^ab^	1.7 ± 0.55 ^bc^	1.0 ± 0.23 ^abc^	1.1 ± 0.19 ^abc^	***	***	***
P6	(*E*)-ligustilide	1764	B [8,10]	0.12 ± 0.02 ^a^	0.15 ± 0.10 ^a^	0.24 ± 0.01 ^a^	0.23 ± 0.03 ^a^	0.25 ± 0.05 ^a^	0.14 ± 0.01 ^a^	0.18 ± 0.09 ^a^	0.18 ± 0.05 ^a^	tr ± 0.02 ^a^	tr ± 0.02 ^a^	0.10 ± 0.03 ^a^	0.11 ± 0.03 ^a^	0.25 ± 0.04 ^a^	tr ± 0.02 ^a^	tr ± 0.01 ^a^	tr ± 0.02 ^a^	*	*	*
	Total			10	16	27	23	22	17	22	21	7.0	5.1	11	18	7.3	9.6	7.3	7.2			
	Aromatic Hydrocarbons																					
AHC1	toluene	769	A	nd ^a^	nd ^a^	nd ^a^	nd ^a^	nd ^a^	nd ^a^	nd ^a^	nd ^a^	0.24 ± 0.11 ^bc^	0.23 ± 0.11 ^bc^	0.38 ± 0.10 ^c^	0.25 ± 0.07 ^bc^	0.17 ± 0.01 ^ab^	0.19 ± 0.04 ^abc^	0.29 ± 0.06 ^bc^	0.27 ± 0.08 ^bc^	***	***	***
AHC2	*p*-xylene	876	B [19]	nd ^a^	nd ^a^	nd ^a^	nd ^a^	nd ^a^	nd ^a^	nd ^a^	nd ^a^	0.11 ± 0.08 ^ab^	0.12 ± 0.06 ^b^	0.14 ± 0.05 ^b^	0.09 ± 0.01 ^ab^	0.11 ± 0.01 ^ab^	0.17 ± 0.05 ^b^	0.15 ± 0.03 ^b^	0.15 ± 0.03 ^b^	***	***	***
	Total			0	0	0	0	0	0	0	0	0.35	0.35	0.52	0.34	0.28	0.36	0.44	0.42			
	Oxides																					
O1	caryophyllene oxide	1610	A	tr ± 0.01 ^abc^	0.13 ± 0.04 ^abcdef^	0.25 ± 0.05 ^cdef^	tr ± 0.02 ^abcd^	0.10 ± 0.07 ^abcde^	0.10 ± 0.02 ^abcde^	tr ± 0.01 ^ab^	nd ^a^	0.25 ± 0.06 ^cdef^	0.27 ± 0.08 ^cdef^	0.28 ± 0.04 ^ef^	0.24 ± 0.09 ^bcdef^	0.26 ± 0.03 ^cdef^	0.33 ± 0.11 ^f^	0.22 ± 0.03 ^abcdef^	0.27 ± 0.11 ^def^	***	***	***
	Lactone																					
L1	γ-nonalactone	1372	A	nd ^a^	nd ^a^	nd ^a^	nd ^a^	nd ^a^	nd ^a^	nd ^a^	nd ^a^	0.10 ± 0.01 ^bcd^	0.10 ± 0.02 ^bcd^	tr ± 0.01 ^abc^	tr ± 0.01 ^ab^	0.10 ± 0.01 ^bcde^	0.10 ± 0.01 ^cde^	0.10 ± 0.03 ^de^	0.10 ± 0.01^e^	***	***	***
L2	dihydroactinolide	1557	B [35]	nd ^a^	nd ^a^	nd ^a^	nd ^a^	nd ^a^	nd ^a^	nd ^a^	nd ^a^	tr ± 0.06 ^ab^	0.10 ± 0.05 ^abc^	0.10 ± 0.02 ^abc^	n.d. ^a^	0.16 ± 0.01 ^c^	0.10 ± 0.06 ^abc^	0.10 ± 0.03 ^bc^	tr ± 0.02 ^ab^	***	***	***
	Total			0	0	0	0	0	0	0	0	0.10	0.13	0.11	0.03	0.32	0.15	0.19	0.13			
	Unknowns																					
U1	unknown 1	n/a		0.57 ± 0.09 ^abc^	0.31 ± 0.03 ^ab^	0.43 ± 0.06 ^ab^	0.19 ± 0.02 ^ab^	0.27 ± 0.01 ^ab^	0.71 ± 0.20 ^bc^	1.2 ± 0.47^c^	0.51 ± 0.29 ^abc^	nd ^a^	nd ^a^	nd ^a^	nd ^a^	nd ^a^	nd ^a^	nd ^a^	nd ^a^	***	***	***
U2	unknown 2	n/a		2.3 ± 0.63 ^bc^	1.7 ± 0.03 ^abc^	2.1 ± 0.06 ^abc^	0.84 ± 0.02 ^ab^	1.0 ± 0.01 ^ab^	2.7 ± 0.20 ^bc^	3.4 ± 0.47 ^c^	1.5 ± 0.29 ^abc^	nd ^a^	nd ^a^	nd ^a^	nd ^a^	nd ^a^	nd ^a^	nd ^a^	nd ^a^	***	***	***
U3	unknown 3	735		nd ^a^	nd ^a^	nd ^a^	nd ^a^	nd ^a^	nd ^a^	nd ^a^	nd ^a^	0.19 ± 0.08 ^b^	0.17 ± 0.05 ^b^	0.25 ± 0.01 ^b^	0.25 ± 0.05 ^b^	0.14 ± 0.01 ^b^	0.16 ± 0.04 ^b^	0.23 ± 0.02 ^b^	0.18 ± 0.03 ^b^	***	***	***
U4	unknown 4	766		nd ^a^	nd ^a^	nd ^a^	nd ^a^	Nd ^a^	Nd ^a^	Nd ^a^	Nd ^a^	0.17 ± 0.08 ^b^	0.15 ± 0.03 ^b^	0.23 ± 0.03 ^b^	0.17 ± 0.01 ^b^	0.12 ± 0.02 ^ab^	0.11 ± 0.09 ^ab^	0.15 ± 0.01 ^b^	0.19 ± 0.02 ^b^	***	***	***
U5	unknown 5	787		nd ^a^	nd ^a^	nd ^a^	nd ^a^	nd ^a^	nd ^a^	nd ^a^	nd ^a^	0.23 ± 0.11 ^b^	0.20 ± 0.07 ^b^	0.23 ± 0.09 ^b^	0.23 ± 0.05 ^b^	0.16 ± 0.02 ^ab^	0.18 ± 0.06 ^ab^	0.28 ± 0.06 ^b^	0.22 ± 0.05 ^b^	***	***	***
U6	unknown 6	896		nd ^a^	nd ^a^	nd ^a^	nd ^a^	nd ^a^	nd ^a^	nd ^a^	nd ^a^	0.22 ± 0.09 ^b^	0.16 ± 0.04 ^b^	0.25 ± 0.07 ^b^	0.22 ± 0.05 ^b^	0.17 ± 0.01 ^b^	0.22 ± 0.03 ^b^	0.22 ± 0.05 ^b^	0.16 ± 0.06 ^b^	***	***	***
U7	unknown 7	971		nd ^a^	nd ^a^	nd ^a^	nd ^a^	nd ^a^	nd ^a^	nd ^a^	nd ^a^	0.64 ± 0.04 ^bc^	0.52 ± 0.06 ^ab^	1.1 ± 0.01 ^c^	0.78 ± 0.17 ^bc^	0.42 ± 0.04 ^ab^	0.58 ± 0.02 ^bc^	0.64 ± 0.05 ^bc^	0.73 ± 0.03 ^b^	***	***	***
U8	unknown 8	1249		nd ^a^	nd ^a^	nd ^a^	nd ^a^	nd ^a^	nd ^a^	nd ^a^	nd ^a^	0.54 ± 0.18 ^b^	0.46 ± 0.06 ^b^	0.65 ± 0.06 ^b^	0.59 ± 0.02 ^b^	0.55 ± 0.03 ^b^	0.56 ± 0.13 ^b^	0.52± 0.05 ^b^	0.49± 0.02 ^b^	***	***	***
U9	unknown 9	1279		0.16 ± 0.06 ^ab^	0.08 ± 0.01 ^a^	0.10 ± 0.01 ^a^	0.13 ± 0.03 ^a^	0.24 ± 0.01 ^ab^	0.11 ± 0.01 ^a^	0.17 ± 0.03 ^ab^	0.10 ± 0.04 ^ab^	0.29 ± 0.12 ^ab^	0.18 ± 0.06 ^ab^	0.19 ± 0.07 ^ab^	0.18 ± 0.02 ^ab^	0.17 ± 0.05 ^ab^	0.22 ± 0.05 ^ab^	0.14 ± 0.04 ^ab^	0.50 ± 0.19 ^bc^	*	*	*
U10	unknown 10	1362		0.10 ± 0.02 ^ab^	0.09 ± 0.03 ^ab^	nd ^a^	0.16 ± 0.01 ^b^	0.03 ± 0.04 ^a^	0.10 ± 0.01 ^ab^	0.08 ± 0.01 ^ab^	0.07 ± 0.4 ^a^	nd ^a^	nd ^a^	nd ^a^	nd ^a^	nd ^a^	nd ^a^	nd ^a^	nd ^a^	***	**	***
U11	unknown 11	1506		nd ^a^	nd ^a^	nd ^a^	nd ^a^	nd ^a^	nd ^a^	nd ^a^	nd ^a^	0.10 ± 0.05 ^ab^	0.10 ± 0.01 ^ab^	0.13 ± 0.04^b^	0.10 ± 0.05 ^ab^	0.10 ± 0.03 ^a^	0.13 ± 0.05 ^b^	0.13 ± 0.03 ^b^	0.13 ± 0.06 ^b^	**	***	***
U12	unknown 12	1539		0.25 ± 0.02 ^ab^	0.33 ± 0.04 ^b^	0.19 ± 0.02 ^ab^	0.13 ± 0.01 ^a^	0.10 ± 0.04 ^ab^	0.10 ± 0.01 ^a^	0.18 ± 0.01 ^ab^	0.12 ± 0.04 ^ab^	0.10 ± 0.04 ^a^	0.10 ± 0.07 ^a^	0.17 ± 0.04 ^ab^	0.20 ± 0.02 ^ab^	0.11 ± 0.02 ^a^	0.17 ± 0.07 ^ab^	0.10 ± 0.01 ^a^	0.13 ± 0.06 ^ab^	**	**	**
U13	unknown 13	1684		nd ^a^	nd ^a^	nd ^a^	nd ^a^	nd ^a^	nd ^a^	nd ^a^	nd ^a^	tr ± 0.06 ^a^	tr ± 0.02 ^a^	tr ± 0.02 ^a^	tr ± 0.03 ^a^	tr ± 0.02 ^a^	0.10 ± 0.01 ^a^	tr ± 0.02 ^a^	tr ± 0.01 ^a^	*	**	*
U14	unknown 14	1706		nd ^a^	nd ^a^	nd ^a^	nd ^a^	nd ^a^	nd ^a^	nd ^a^	nd ^a^	0.10 ± 0.09 ^ab^	tr ± 0.02 ^ab^	0.10 ± 0.02 ^ab^	0.11 ± 0.01 ^b^	0.10 ± 0.04 ^ab^	0.13 ± 0.02^b^	0.10 ± 0.03 ^ab^	0.10 ± 0.05 ^ab^	***	***	***
U15	unknown 15	1799		nd ^a^	nd ^a^	nd ^a^	nd ^a^	nd ^a^	nd ^a^	nd ^a^	nd ^a^	0.13 ± 0.03 ^b^	0.13 ± 0.05 ^b^	0.18 ± 0.01 ^b^	0.13 ± 0.04 ^b^	0.10 ± 0.01 ^b^	0.18 ± 0.04 ^b^	0.12 ± 0.02 ^b^	0.13 ± 0.05 ^b^	***	***	***
	Total			3.4	2.5	2.9	1.4	1.8	3.8	5.1	2.4	2.7	2.2	3.5	3.0	2.2	2.7	2.6	3.0			

^A^ Linear retention index on a H*P*-5MS column. ^B^ A, mass spectrum and LRI agree with those of authentic compounds; B, mass spectrum (spectral quality value >80 was used); LRI agrees with reference spectrum in the NIST/EPA/NIH mass spectra database and LRI agrees with those in the literature cited; $ tentatively identified, spectral quality value of 70 was used for this compound. ^C^ Percentage composition of total peak area divided by compound peak area; means labelled with letters are significantly different (*p* < 0.05) according to the GxE interaction; means of three replicate samples; tr, trace amounts <0.10%; nd, not detected. ^D^ Probability, obtained by ANOVA, that there is a difference between means; ns, no significant difference between means (*p* > 0.05); * significant at the 5% level; ** significant at the 1% level; *** significant at 0.1% level. ^E^ Geographical location. ^F^ Genotype. ^G^ Geographical location x genotype interaction. Cells are colour coded; orange expresses the location giving the higher value for each compound for each genotype; green expresses the location giving the lower value of each compound for each genotype; no colour expresses no difference in percentage composition for both locations.

**Table 2:** Mean panel scores for sensory attributes of the eight celery samples harvested in UK 2018 and Spain 2019.

Attribute	Score ^A^																	
	UK								P ^B^	Spain								P ^B^
	5	8	10	12	15	18	22	25		5	8	10	12	15	18	22	25	
Appearance																		
Colour	56.4 ^b^	63.6 ^ab^	62.6 ^ab^	72.9 ^a^	72.1 ^a^	65.6 ^ab^	70.5 ^a^	26.8 ^c^	***	45.6 ^c^	51.2 ^c^	50.0 ^c^	69.9 ^ab^	71.8 ^a^	56.0 ^bc^	71.6 ^a^	26.7 ^d^	***
Stalk thickness	49.8 ^ab^	49.5 ^ab^	55.8 ^a^	20.9 ^b^	58.7 ^a^	62.5 ^a^	61.3 ^a^	55.0 ^a^	***	42.4 ^ab^	46.8 ^ab^	38.2 ^bc^	27.3 ^c^	55.5 ^a^	55.9 ^a^	58.4 ^a^	54.4 ^a^	***
Ribbed	46.6 ^bc^	61.0 ^ab^	61.7 ^a^	65.9 ^a^	35.5 ^cd^	25.4 ^d^	34.2 ^cd^	37.4 ^cd^	***	66.7 ^a^	64.0 ^ab^	67.9 ^a^	76.1 ^a^	48.4 ^c^	42.1 ^c^	49.6 ^bc^	49.5 ^bc^	***
Odour																		
Fresh fennel	16.5	14.2	18.9	15.5	15.3	18.6	15.4	18.2	ns	19.5	18.4	16.8	15.4	24.8	19.9	15.8	13.7	ns
Grassy/green	32.6 ^a^	31.0 ^ab^	32.1 ^ab^	36.3 ^a^	30.7 ^ab^	28.3 ^ab^	35.3 ^a^	21.1 ^b^	***	11.6 ^b^	19.4 ^ab^	24.3 ^a^	25.6 ^a^	23.5 ^a^	20.1 ^ab^	23.2 ^a^	19.2 ^ab^	**
Fresh parsley	14.1	19.7	19.0	19.1	20.6	16.7	16.7	10.8	ns	11.5	15.5	16.8	16.1	18.5	16.6	14.1	11.4	ns
Fresh coriander	12.8	12.1	14.2	11.7	14.2	17.5	15.4	11.1	ns	17.9	18.9	21.5	15.1	22.8	22.7	17.7	14.3	ns
Taste/flavour																		
Bitter	23.1 ^abc^	24.0 ^abc^	24.7 ^abc^	35.9 ^a^	28.2 ^abc^	31.3 ^ab^	24.4 ^abc^	15.5 ^c^	ns	24.4 ^ab^	30.9 ^ab^	29.4 ^ab^	30.9 ^ab^	28.4 ^ab^	36.4 ^a^	26.1 ^ab^	18.1 ^b^	**
Salt	nd	nd	nd	nd	nd	nd	nd	nd	**	26.4	22.6	27.3	31.3	23.4	31.2	24.8	18.7	ns
Sweet	15.2 ^bcd^	20.3 ^ab^	21.6 ^ab^	10.6 ^d^	15.6 ^bcd^	12.2 ^cd^	20.0 ^ab^	24.6 ^a^	***	18.3	19.8	21.4	18.2	20.0	14.5	16.1	22.8	ns
Fresh fennel	11.9	10.3	12.6	11.0	7.7	13.6	11.6	11.3	ns	15.0	15.7	10.4	13.2	17.4	13.6	8.0	10.8	ns
Rocket	11.3 ^bc^	13.4 ^bc^	12.4 ^bc^	23.8 ^a^	16.6 ^abc^	16.9 ^abc^	10.4 ^bc^	7.7 ^c^	***	1.8	2.0	3.2	1.8	1.4	1.0	0.8	0.2	ns
Fresh coriander	17.5	16.3	16.0	9.6	15.0	18.1	18.9	14.1	ns	17.2	21.0	18.1	17.4	18.0	21.4	15.7	13.8	ns
Soapy	18.2 ^ab^	12.4 ^b^	16.4 ^ab^	18.4 ^ab^	15.4 ^ab^	23.7 ^a^	16.3 ^ab^	13.0 ^ab^	*	19.1	20.5	25.1	22.0	20.0	27.5	19.7	15.0	ns
Cucumber	25.7 ^ab^	33.2 ^ab^	30.4 ^ab^	9.1 ^c^	30.0 ^ab^	22.4 ^b^	27.9 ^ab^	37.7 ^a^	***	12.8	14.1	9.9	5.8	15.3	11.8	11.8	14.8	ns
Mouthfeel																		
Crunchy	65.4 ^abc^	62.6 ^bc^	64.9 ^abc^	56.7 ^c^	70.2 ^ab^	66.4 ^abc^	73.7 ^a^	62.5 ^bc^	***	64.0	67.4	67.8	61.9	70.5	66.2	70.3	65.5	ns
Stringy	40.8 ^b^	46.6 ^b^	40.1 ^b^	64.1 ^a^	33.2 ^b^	40.6 ^b^	35.1 ^b^	35.2 ^b^	***	60.2 ^ab^	58.2 ^ab^	59.9 ^ab^	71.9 ^a^	47.2 ^bc^	57.3 ^abc^	38.5 ^c^	52.4 ^abc^	***
Moist	50.6 ^a^	47.2 ^a^	50.0 ^a^	29.7 ^b^	53.1 ^a^	44.3 ^a^	51.4 ^a^	54.8 ^a^	***	49.9	55.8	45.1	35.5	58.6	47.8	52.1	56.2	ns
Firmness of first bite	63.7	59.9	63.3	59.2	68.9	65.7	67.6	58.6	ns	64.8	66.1	65.6	63.5	67.2	63.2	69.9	63.2	ns
Aftereffects																		
Numbness	13.1	8.6	13.8	11.5	10.0	14.0	9.8	9.0		17.0	19.3	20.9	16.4	21.1	23.1	16.0	11.4	ns
Bitter	17.4 ^bc^	18.4 ^bc^	18.3 ^bc^	29.0 ^a^	19.1 ^bc^	25.7 ^ab^	16.0 ^bc^	12.0 ^c^	***	16.7 ^ab^	19.4 ^ab^	24.3 ^a^	21.8 ^ab^	19.2 ^ab^	25.0 ^a^	17.2 ^ab^	12.0 ^b^	*
Soapy	16.9 ^ab^	15.7 ^ab^	16.7 ^ab^	21.2 ^ab^	19.9 ^ab^	24.8 ^a^	18.6 ^ab^	12.9 ^b^	*	18.3	21.5	22.7	20.8	21.7	25.5	18.8	11.7	ns
Grassy/green	27.7	27.0	30.3	27.6	28.4	26.4	31.4	19.0	ns	12.3	13.3	15.8	19.9	15.8	14.3	15.7	13.6	ns

^A^ Means are from two replicate samples; differing small letters (a, b, c, d, e, f) represent sample significance from multiple comparisons and means not labelled with the same letters are significantly different (*p* < 0.05); nd, not detected. ^B^ Probability obtained by ANOVA that there is a difference between means; ns, no significant difference between means (*p* > 0.05); * significant at the 5% level; ** significant at the 1% level; *** significant at 0.1% level.

**Table 3:** Environmental data recorded at the nearest weather station to the farm of growth and provided by G’s Fresh (UK) and Grupo G’s España.

	Ely, Cambridgeshire (UK)					Aguilas, Mercia (Spain)				
Weeks after Transplant	Air Temp (°C)	Rainfall (mm)	Relative Humidity (%)	Wind Speed (m/s)	Dew Point (°C)	Air Temp (°C)	Rainfall (mm)	Relative Humidity (%)	Wind Speed (m/s)	Dew Point (°C)
1	17.0	0.0	73.0	2.4	15.4	15.3	0.0	79.6	0.8	1.9
2	14.7	0.0	81.3	1.5	18.7	15.4	0.1	76.3	1.1	3.9
3	16.4	0.1	66.1	1.3	20.0	19.9	0.0	72.8	2.4	4.1
4	17.0	0.0	94.8	1.6	18.4	17.4	0.1	63.7	2.9	1.1
5	18.9	0.0	98.5	1.5	20.4	16.9	0.0	82.1	1.0	6.9
6	19.8	0.0	99.7	3.0	16.3	16.4	0.0	81.2	1.9	6.1
7	18.2	0.0	99.4	1.4	6.5	16.6	0.0	82.5	1.2	6.3
8	20.4	0.0	99.0	1.9	16.3	18.5	0.0	84.7	0.8	8.2
9	21.4	0.1	70.5	2.1	18.2	18.9	0.0	78.3	1.3	6.9
10	20.9	0.0	71.8	2.6	13.9	19.8	0.0	79.4	1.4	7.2
11	17.3	0.2	99.9	1.0	12.4	17.9	0.3	71.1	2.2	5.1
12	18.4	0.0	98.6	2.3	12.9	16.9	1.8	78.3	2.1	8.0
13	15.8	0.0	93.9	2.0	12.4	19.0	0.6	74.3	2.4	6.6
Average	18.2	0.0	88.1	1.9	15.5	17.6	0.4	77.3	1.7	6.0

## Data Availability

The data presented in this study are available upon request from the corresponding author.

## Supplementary Materials

The following are available online at https://www.mdpi.com/article/10.3390/ijms222112016/s1.
