# Supplementary material for: Investigating the Relationship of Genotype and Geographical Location on Volatile Composition and Sensory Profile of Celery (Apium graveolens)
Source: Int J Mol Sci. 2021 Nov 6;22(21):12016. doi: 10.3390/ijms222112016 (PMC8584909; doi:10.3390/ijms222112016)
Supplement: Supplementary file 1 [file ijms-22-12016-s001.zip › ijms-1429086-supplementary.pdf]

Table S1: Origin and images of the eight celery samples used in this study and harvested in Ely, United Kingdom and Aguilas, Spain.

Line	Origin	Harvest UK	Harvest Spain	Phenotypic Traits
5	USA	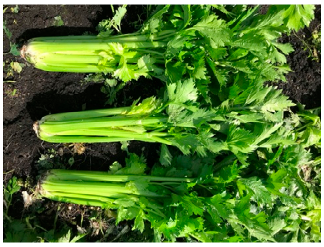	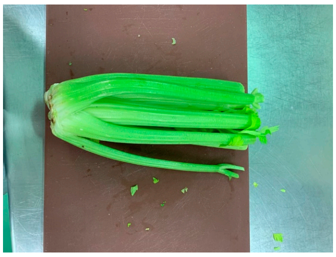	Wide base
8	AUS	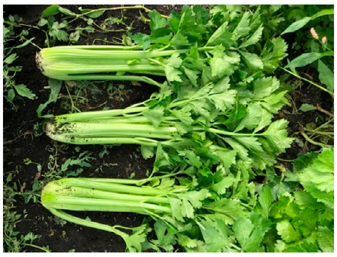	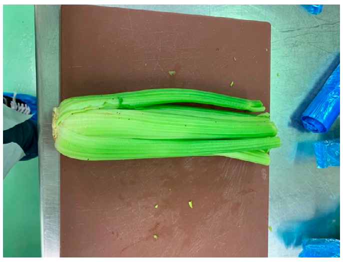	Compact petioles
10	UK	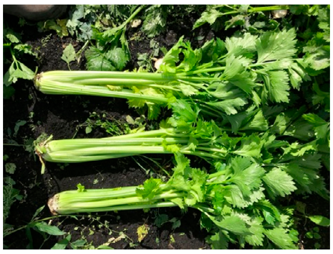	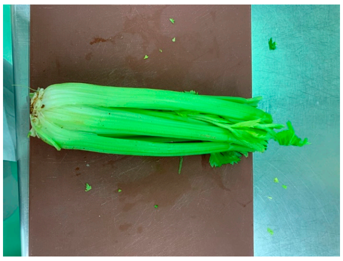	Compact, thin petioles
12	UK	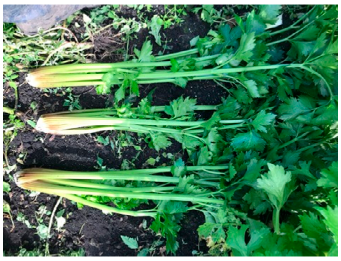	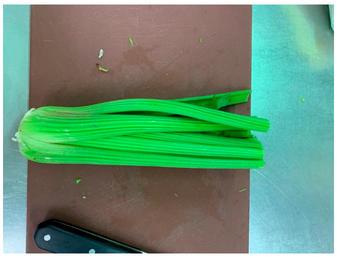	Pink-coloured petioles, present at the base of the plant. Very prominent ribs

15	USA	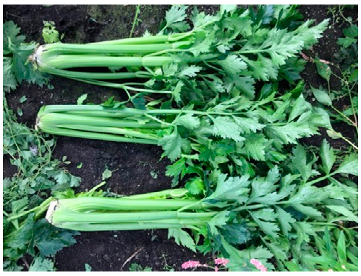	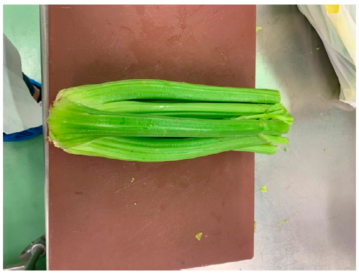	Very dark green outer petioles
18	EU	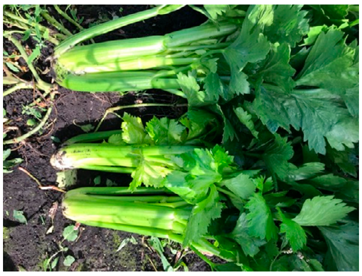	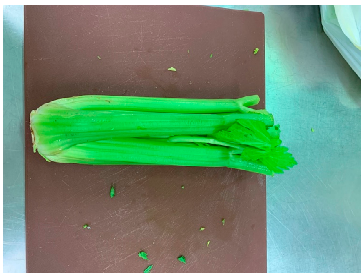	Very short and wide petioles
22	USA	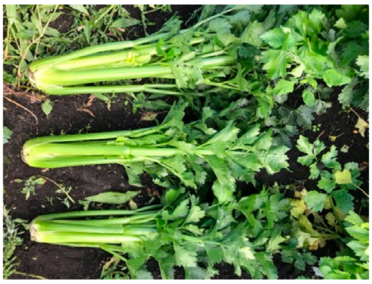	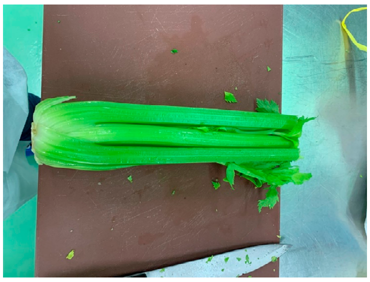	Less prominent ribs, compact petioles
25	EU	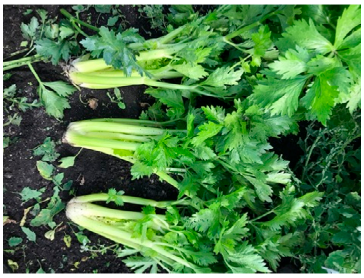	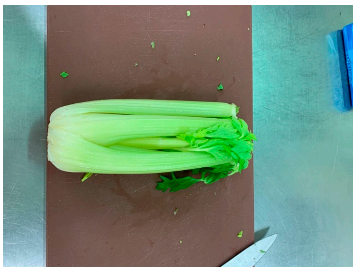	Predominantly white genotype with very short petioles
